# Zwitterionic Polymer-Gated Au@TiO_2_ Core-Shell Nanoparticles for Imaging-Guided Combined Cancer Therapy

**DOI:** 10.7150/thno.35418

**Published:** 2019-07-09

**Authors:** Tao Zheng, Wentao Wang, Fan Wu, Ming Zhang, Jian Shen, Yi Sun

**Affiliations:** 1 Department of Health Technology, Technical University of Denmark, Kongens Lyngby, 2800, Denmark;; 2 Jiangsu Collaborative Innovation Center for Biomedical Functional Materials, School of Chemistry and Materials Science, Nanjing Normal University, Nanjing 210023, P. R. China.

**Keywords:** Au@TiO_2_ core-shell NPs, photothermal, photodynamic, cationic therapy, chemotherapy, magnetic resonance imaging

## Abstract

With advances in nanoparticle (NP) synthesis and engineering, nanoscale agents with both therapeutic and diagnostic functions have been increasingly exploited for cancer management. Herein, we synthesized a new type of zwitterionic polymer-gated Au@TiO_2_ core-shell nanoparticles, which showed that they could selectively target and efficiently eliminate cancer cells via photothermal therapy (PTT), photodynamic therapy (PDT), pH/NIR-induced drug release, and cationic therapy.

**Methods**: In the present study, the multifunctional therapeutic agent [Mn@P(CitAPDMAEMA)@Au@TiO_2_@DOX] was prepared to treat cancer with imaging-guided combination method. Firstly, Au@TiO_2_ core-shell nanoparticles (NPs) were synthesized. Taking advantage of broad and strong photoabsorption and reactive oxygen species (ROS) generation, Au@TiO_2_ core-shell NPs facilitated the single light-induced PTT and PDT. Next, a chemotherapy drug doxorubicin (DOX) was loaded into Au@TiO_2_ core-shell NPs. Then, a biocompatible zwitterionic polymer P(CitAPDMAEMA) was grafted to improve the hemocompatibility of NPs and prolong the circulation time. The polymer also served as a capping or switching material for pH-triggered drug release. In addition, the cationic nature of P(CitAPDMAEMA) eased the binding to human cervical cancer (HeLa) cells and effectively inhibited their growth in acidic environments (termed cationic therapy). Moreover, with Mn^2+^ ions immanently chelated, Mn@P(CitAPDMAEMA)@Au@TiO_2_@DOX NPs were able to provide enhanced contrast under *T*_1_- or *T*_2_-weighted magnetic resonance imaging (MRI).

**Results**: The* in vitro* and* in vivo* anticancer experiments demonstrated the tumor was effectively inhibited with minimal side effects by the multifunctional NPs.

**Conclusions**: As far as we know, this is the first presentation of four therapeutic methods into one nanomaterial, which will open up a new dimension for the design of combined treatment.

## Introduction

Traditional cancer treatment such as chemotherapy can lead to serious side effects, as the anticancer drugs kill both cancerous and normal cells due to the lack of cell specificity 1-5. In the last few years, nanomaterial-based therapeutic methods such as photothermal therapy (PTT), photodynamic therapy (PDT) and controlled drug delivery system have attracted a lot of attention. PTT ablates malignant tissues by utilizing external light-induced hyperthermia. It causes negligible damage to normal tissues, and is convenient, non-invasive, remote-controllable and safe 6-8. Various photothermal agents such as Au nanoparticles (AuNPs) 9, copper NPs 10, carbon derivatives 11, transition metal sulfides 12, black titania 13, and black phosphorus (BPs) 14 have been intensively explored. Among them, AuNPs are preferred due to their high absorption ability in the near-infrared (NIR) region and excellent biocompatibility 9. However, for AuNPs to be good PTT agents, their localized surface plasmon resonance (LSPR) absorption must be tuned to the 550-900 nm region to allow for NIR laser treatment. This could be done by carefully controlling the size and shape of AuNPs, but the synthesis process was complicated. Alternatively, the LSPR coupling between AuNPs and semiconductor has been proposed as a novel approach 15. Interestingly, we found that the titania (TiO_2_) shell modified with AuNPs is one of the most effective methods to shift their LSPR absorption to a longer wavelength. The condensed free-space optical field within the sub-wavelength regions adjacent to the surface of the AuNPs enables significant electric field enhancement under resonant excitation, which increases the efficiency of photothermal conversion.

On the other hand, PDT relies on photosensitizers (PSs) to release the reactive oxygen species (ROS) and cytotoxic free radicals when they absorb light at appropriate wavelengths 16-18. Organic PSs are the most widely used PS types. They mainly release singlet oxygen (^1^O_2_) which is produced by spin inversion of triplet oxygen to singlet oxygen (type II PDT). Some special organic PSs can also form hydrogen peroxide (H_2_O_2_) and hydroxyl radicals (·OH) by ionization events (type I PDT) 19,20. Despite these advantages, the organic PSs depend on oxygen for cytotoxic ROS production, which limits PDT effectiveness in hypoxic conditions, such as many solid tumors 21. Moreover, *in-situ* oxygen in PDT is rapidly depleted, creating transient hypoxia microenvironment, which further reduces the therapeutic effects 22. Consequently, it is necessary to design simple and biocompatible PSs that can evolve oxygen continuously without exogenous activation 23. The semiconductor material TiO_2_ has been considered as a new type of PS, as they are able to catalyze H_2_O_2_ into O_2_. It is reported that H_2_O_2_ is abundant in cancer microenvironment and is an appropriate source for the production of O_2_ within tumors. However, the application of TiO_2_ as PSs has been hampered by the requirement of UV light excitation and the low quantum efficiency 24. In this study, we innovatively combined TiO_2_ with AuNPs to create a novel Au@TiO_2_ core-shell nanohybrid. Coupling TiO_2_ with AuNPs results in enhanced electron-hole separation under red light irradiation, which enables the utilization of PTT and PDT for cancer treatment at NIR region.

The *in vivo* applications of NPs are often hindered by the challenges such as short circulation time, and biological protein adsorption. One effective way to address these problems is to coat the NPs with appropriate polymers. Recently, zwitterionic polymers have attracted a lot of attention as stealth materials in drug delivery systems. Conjugates of zwitterionic polymers with therapeutic agents (i.e. PSs, drugs or proteins) have been demonstrated 25, 26. Due to their limited interactions with biological proteins 27, 28, they showed excellent biocompatibility and could escape from the reticuloendothelial system, achieving long circulation time 30, 31. We synthesized a pH-sensitive polymer with quaternary ammonium groups according to a previous method 32, which was zwitterionic at physiological pH and was converted to a cationic species in acidic environments. Herein, we loaded doxorubicin (DOX) into the Au@TiO_2_ core-shell, then grafted the zwitterionic polymer onto the NP for combined cancer therapy. As the cancer cells' metabolism and the immune response contribute to an acidity tumor microenvironment 33, 34, the zwitterionic polymers convert to a cationic state when in proximity of the tumor tissues. The cationic nature of the polymer, not only enhances cellular uptake due to the strengthened NP-cellular membrane interactions, but also inhibits the cell growth. In addition, the pH-sensitive charge-conversion results in the falling of the polymer chains, making it possible for “on-demand” drug release at the acidic tumor sites.

**Scheme 1:**
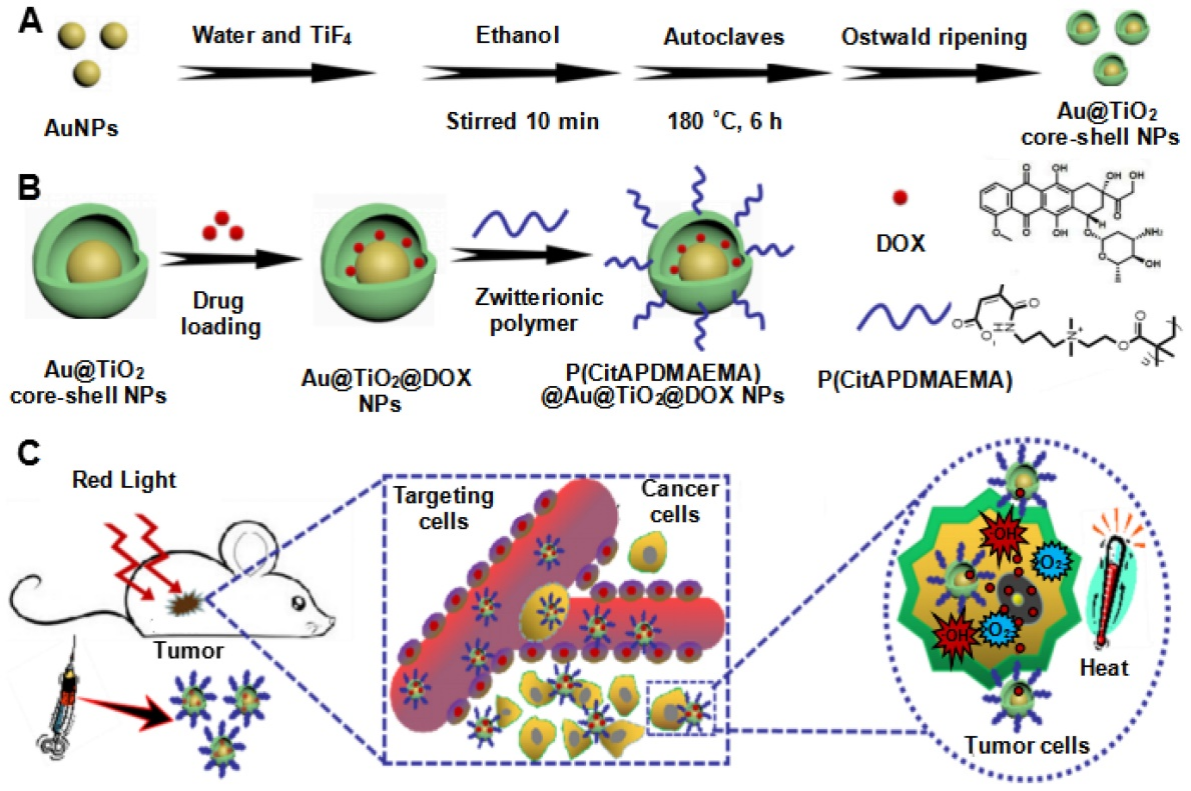
Schematic illustration of the synthesis process of the (A) Au@TiO_2_ core-shell NPs and (B) P(CitAPDMAEMA)@Au@TiO_2_@DOX. (C) Schematic representation of P(CitAPDMAEMA)@Au@TiO_2_@DOX-based delivery system enhancing the cationic therapy, chemotherapy, PDT, and PTT under red-light irradiation.

In the present study, a novel, multifunctional DOX-loaded, manganese ion (Mn^2+^)-chelated complex, and zwitterionic polymer [P(CitAPDMAEMA)]-grafted Au@TiO_2_ ore-shell NPs [Mn@P(CitAPDMAEMA)@Au@TiO_2_@DOX] was reported, which could selectively target and eliminate cancer cells via PTT, PDT, pH/NIR-induced drug release and cationic therapy (Scheme 1). We have synthesized a new Au@TiO_2_ core-shell through *in-situ* growth and the Ostwald ripening process of TiO_2_ shells on the preformed AuNPs (Scheme 1A). The DOX as a chemotherapeutic agent was loaded to the Au@TiO_2_ core-shell NPs, which was then coated by the zwitterionic polymer P(CitAPDMAEMA) (Scheme 1B). We also chelated Mn^2+^ to offer an obvious contrast in *T*_1_-weighted MRI. The synergetic cancer therapy could be achieved under 635 nm laser irradiation (Scheme 1C). Au@TiO_2_ core-shell NPs had both photothermal and photodynamic effects with high ROS generating efficiency and photothermal conversion efficiency. The Au@TiO_2_ core-shell NPs exhibited improved photodynamic performance compared to those of commercial TiO_2_, Au/TiO_2_ hybrid, or Au@TiO_2_ core-shell NPs. The biocompatible P(CitAPDMAEMA) improved the hemocompatibility of the NPs. Meanwhile, the cationic nature of P(CitAPDMAEMA) facilitated the binding to human cervical cancer (HeLa) cells and inhibited their growth effectively in an acidic environment (Scheme S1). P(CitAPDMAEMA) also served as capping or switching materials for Au@TiO_2_@DOX NPs. The drug was only released when the NPs reached the low pH environment and red light irradiation. *In vitro* and *in vivo* studies demonstrated that the Mn@P(CitAPDMAEMA)@Au@TiO_2_@DOX NPs could be used as superior theranostic agents for multimodality image-guided synergistic cancer therapy.

## Results and Discussion

### Preparation and Characterization of Au@TiO_2_ Core-shell NPs

**Figure 1:**
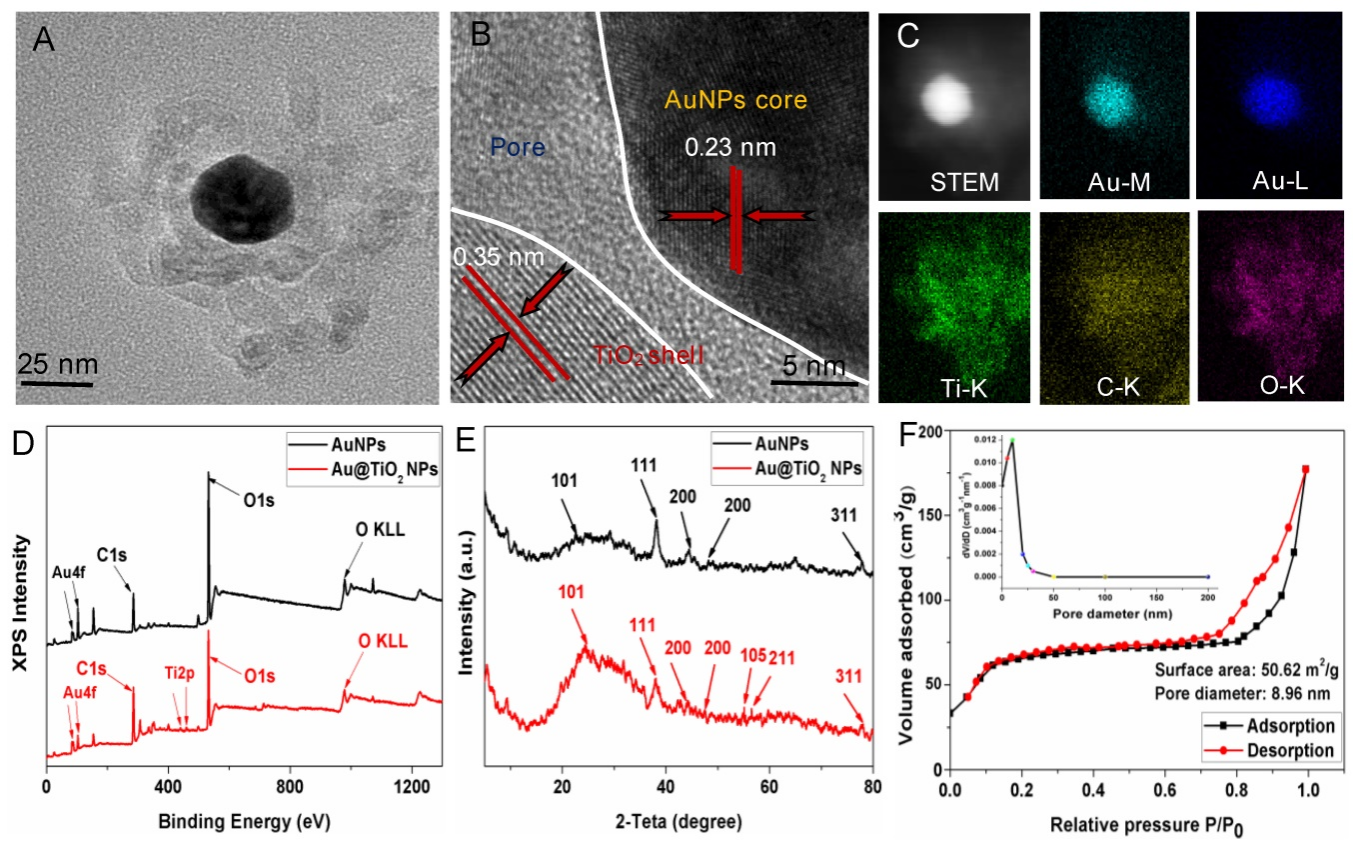
(A) TEM and (B) HRTEM images of Au@TiO_2_ core-shell NPs. (C) STEM image and corresponding two-dimensional elemental maps of Au@TiO_2_ core-shell NPs. (D) XPS survey scan for AuNPs and Au@TiO_2_ core-shell NPs. (E) XRD spectra of AuNPs and Au@TiO_2_ core-shell NPs. (F) N_2_ adsorption-desorption isotherms and pore size distribution (inset) of Au@TiO_2_ core-shell NPs.

The Au@TiO_2_ core-shell NPs were prepared according to previous methods with some modification 35. The overall procedure is shown in Scheme S2. The micro-morphology of the AuNPs (Figure S1A) and Au@TiO_2_ core-shell NPs (Figure S1B) were studied by transmission electron microscopy (TEM). Figure 1A shows the high-resolution TEM (HR-TEM) images of Au@TiO_2_ core-shell NPs. Due to the differential electron densities of Au and TiO_2_, the darker spots were the Au core while lighter areas were TiO_2_ shells. It is worth noting that AuNPs were surrounded by a TiO_2_ shell. Furthermore, the hollow space between the core and the shell in each nanohybrid could be observed clearly. The Au core could move within the TiO_2_ shell, producing an eccentric shape of the Au@TiO_2_ core-shell NPs. The Au@TiO_2_ core-shell NPs exhibited a uniform diameter of 108 nm (Figure S2A), with an average Au core size of 30 ± 1.7 nm and an average TiO_2_ shell thickness of 32 ± 3.5 nm. The dynamic light scattering (DLS) results indicated that the average hydrodynamic particle size of Au@TiO_2_ core-shell NPs was 135 ± 3.8 nm (Figure S2B), revealing that they were stable in deionized water without aggregation. HR-TEM images revealed that the TiO_2_ shell was polycrystalline with many small and randomly oriented crystallites. The lattice fringes with a d-spacing of 0.23 nm could be indexed to the (111) plane of Au (Figure 1B), whereas the d-spacing of 0.35 nm agreed with the (101) plane of TiO_2_ 35. Moreover, Energy-dispersive X-ray (EDS) elemental mapping was also applied to characterize the functionalization of Au@TiO_2_ core-shell NPs. Figure 1C shows the uniform elemental distribution (Ti, Au, and O). The Ti element mapping was derived from the TiO_2_ layer and the Au element mapping, suggesting the existence of AuNPs.

The components of the Au@TiO_2_ core-shell structure were studied by X-ray photoelectron spectroscopy (XPS). Figure 1D shows that the Au 4f doublet peaks appeared at 83.3 and 86.9 eV 35, where the splitting of 4.5 eV further indicated the metallic nature of Au (Figure S3A). The Ti 2p peaks for TiO_2_ were located at about 458.4 and 464.2 eV with the splitting of the 2p doublet being at 5.8 eV (Figure 1D and Figure S3B), confirming a Ti^4+^ state in octahedral coordination with oxygen 36. The XPS spectra peak of O 1 s at 532.1 eV was attributed to the Ti-O bonds in the TiO_2_ lattice (Figure S3C). In addition, X-ray diffraction (XRD) was used to study two different crystal nanostructures. Figure 1E shows that all the samples displayed two series of peaks, which belongs to the anatase-TiO_2_ structure and the face-centered-cubic Au. The peaks at 2θ of 25.3, 47.9, 53.8, and 54.7 could be ascribed to the (101), (200), (105), and (211) planes of anatase-TiO_2_ 37, respectively, while the left peaks were corresponding to the (111), (200), (220), and (311) faces of face-centered-cubic Au. Thus, the presence of Au and TiO_2_ in the Au@TiO_2_ core-shell NPs were confirmed.

Nitrogen physisorption measurements were also used to study the properties of the Au@TiO_2_ core-shell NPs structure. Figure 1F displays the N_2_ adsorption-desorption isotherms and the respective pore size distribution curves of samples. The Au@TiO_2_ core-shell NPs displayed a type IV isotherm with a well-defined hysteresis loop, which indicated well-developed mesoporous characteristics. The BET surface areas of the Au@TiO_2_ core-shell NPs were estimated to be 50.62 m^2^/g, and its average pore diameters were about 8.96 nm, indicating that the Au@TiO_2_ core-shell NPs could provide more active sites and easier passage for the drug into the shell.

### Drug Loading

**Figure 2:**
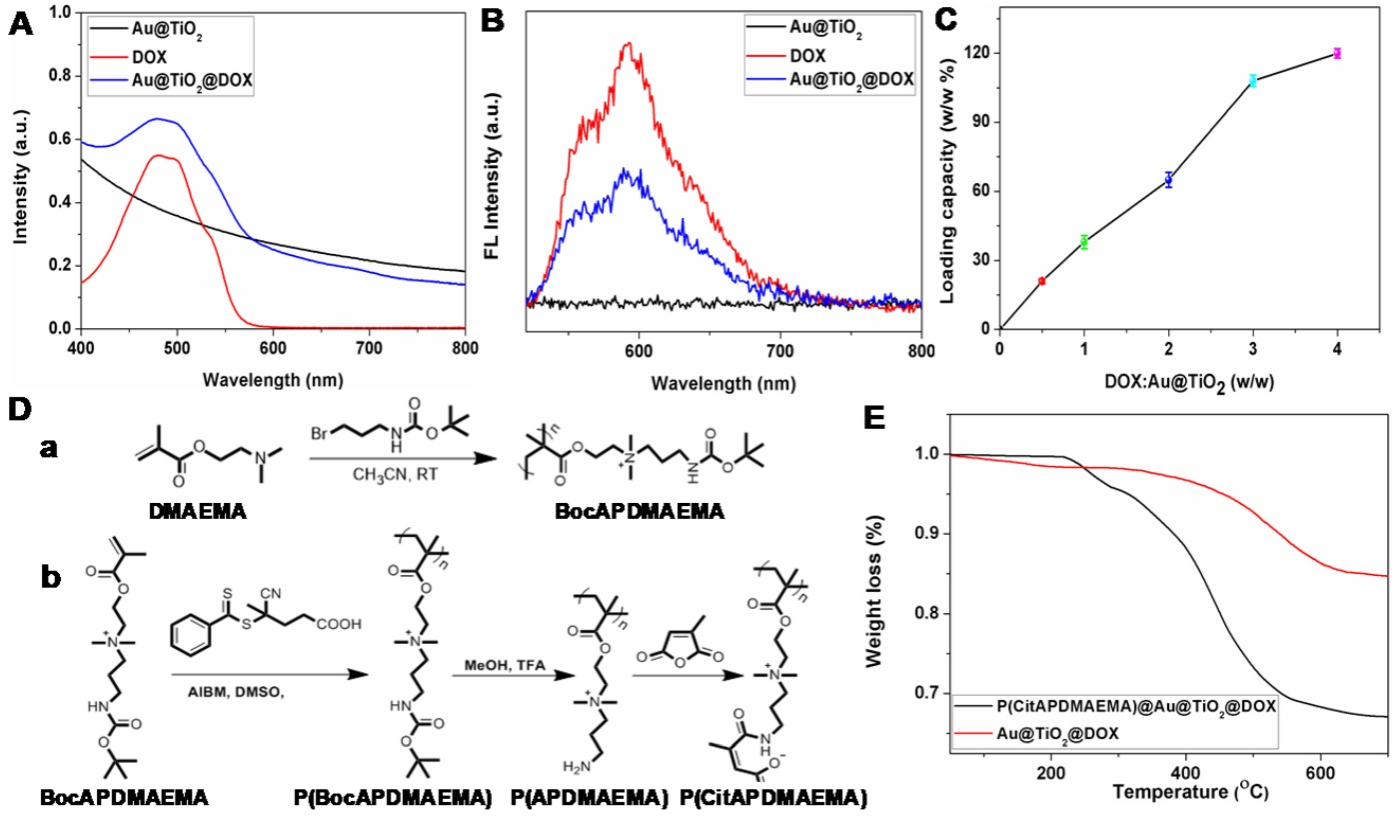
(A) UV-vis and (B) fluorescence spectra of Au@TiO_2_ core-shell NPs, DOX, and Au@TiO_2_@DOX NPs. (C) Quantification of DOX loading at different DOX: Au@TiO_2_ core-shell NPs ratios. (n = 3, mean ± s.d.) (D) Synthetic route for the preparation of (a) BocAPDMAEMA and (b) P(CitAPDMAEMA). (E) TGA curves of Au@TiO_2_@DOX NPs and P(CitAPDMAEMA)@Au@TiO_2_@DOX NPs.

In this study, we selected DOX as the model drug to test the feasibility of Au@TiO_2_ core-shell NPs as a drug delivery carrier. The conjugation was confirmed by zeta potential, UV-vis spectra and fluorescence spectra 38. The zeta potential of Au@TiO_2_ core-shell NPs changed from -18.9 to -1.2 mV (Figure S4A-B) after DOX loading, indicating that the negatively charged Au@TiO_2_ core-shell NPs bonded with positively charged DOX (Figure S4C). A notable difference of Au@TiO_2_@DOX core-shell NPs was found in the spectra where the UV-vis absorption peak located at 490 nm (Figure 2A). It was caused by the absorption of DOX. Moreover, the fluorescence of DOX was quenched after DOX loading on the Au@TiO_2_ core-shell NPs (Figure 2B), verifying an interaction between the two components. By increasing the weight ratio (feeding DOX/Au@TiO_2_ core-shell NPs), the drug loaded on the NPs also increased (Figure 2C). The maximum loading capacity reached ~120%. There are two factors contributing to the high drug-loading capacity of the Au@TiO_2_ core-shell NPs. One is that, the Au@TiO_2_ core-shell NPs could bind to DOX molecules via different types of interactions, such as electrostatic attraction and hydrogen bonding. The other reason is that the unique structure of the Au@TiO_2_ core-shell NPs with a large central hollow cavity provided enough space for loading the drug. Thus, the distributed DOX molecules could be retained and kept in the Au@TiO_2_ carriers to form DOX-loaded Au@TiO_2_ NPs.

### Synthesis of P(CitAPDMAEMA@Au@TiO_2_@DOX NPs

As mentioned previously, it is desirable to develop high-performance biocompatible anticancer agents for cancer treatment, it is necessary to cap or switch nanocarriers with porous structures 39. In light of that, the smart pH-sensitive polymer P(CitAPDMAEMA) was synthesized 29, as shown in Figure 2D. Characterization of these materials was shown in Figure S5. P(CitAPDMAEMA) was coated to the surface of Au@TiO_2_@DOX NPs, through a physicochemical interactions, such as π-π stacking, hydrophobic and electrostatic interactions. The Fourier transform infrared (FT-IR) spectra (Figure S6) of P(CitAPDMAEMA)@Au@TiO_2_@DOX NPs confirmed the efficient surface modification of P(CitAPDMAEMA) on the Au@TiO_2_@DOX NPs 39. The resultant P(CitAPDMAEMA)@Au@TiO_2_@DOX NPs showed no remarkable changes in morphology (Figure S7A) as compared to Au@TiO_2_ NPs. Compared to Au@TiO_2_ NPs, the larger diameter of P(CitAPDMAEMA)@Au@TiO_2_@DOX NPs was likely due to the presence of P(CitAPDMAEMA) loaded on the surface of the Au@TiO_2_@DOX NPs (Figure S7B). As illustrated in Figure S8A, the P(CitAPDMAEMA)@Au@TiO_2_@DOX NPs exhibited high stability in phosphate buffered solution (PBS), fetal bovine serum (FBS), and Dulbecco's modified Eagle medium (DMEM).

The P(CitAPDMAEMA) content in the P(CitAPDMAEMA)@Au@TiO_2_@DOX NPs was evaluated by thermal gravimetric analysis (TGA) (Figure 2E). The different weight loss between the P(CitAPDMAEMA)@Au@TiO_2_@DOX NPs and the Au@TiO_2_@DOX NPs at 700 °C indicated that the P(CitAPDMAEMA) grafting amount was about 18.4%. Full nitrogen adsorption isotherms (Figure S8B) were observed in order to obtain information about the specific surface areas of the P(CitAPDMAEMA)@Au@TiO_2_@DOX NPs. The BET surface area and the total pore volume of the P(CitAPDMAEMA)@Au@TiO_2_@DOX NPs were found to be 9.34 m^2^/g and 0.112 cm^3^/g, respectively, which were smaller than those of the Au@TiO_2_@DOX NPs (41.17 m^2^/g and 0.674 cm^3^/g, respectively)_._ These results indicated that P(CitAPDMAEMA)@Au@TiO_2_@DOX NPs not only showed a considerable absence of porosity but also had important pore blocking because of the valve-like function of P(CitAPDMAEMA).

### Photothermal Properties of P(CitAPDMAEMA)@Au@TiO_2_@DOX NPs

**Figure 3:**
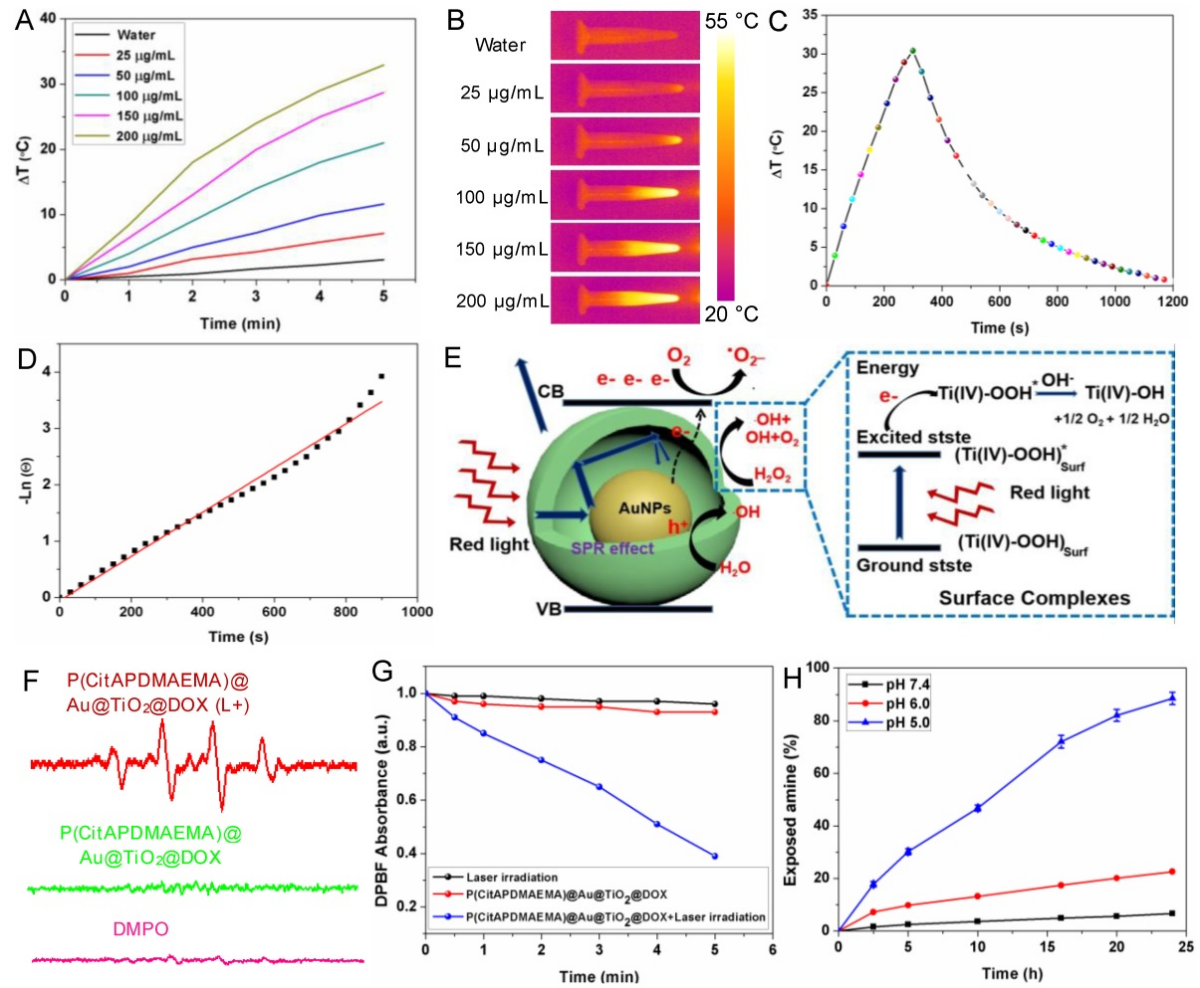
(A) Temperature curves of P(CitAPDMAEMA)@Au@TiO_2_@DOX NPs at different concentrations. (B) IR images of water and P(CitAPDMAEMA)@Au@TiO_2_@DOX NPs solutions under 635 nm laser irradiation (2.0 W/cm^2^, 5 min). (C) Photothermal effect of P(CitAPDMAEMA)@Au@TiO_2_@DOX NPs solution (200 µg/mL) under 635 nm laser irradiation (2.0 W/cm^2^, 5 min). (D) Linear relationship between time and Ln (θ) obtained from the cooling time of (C). (E) Mechanism illustration for the enhanced photodynamic activity of Au@TiO_2_ core-shell NPs. The hollow structure could increase the optical path through multiple reflections and scattering, which enhanced light capturing inside the core-shell NPs and boosts light utilization. (F) EPR spectra (DMPO/·OH) of P(CitAPDMAEMA)@Au@TiO_2_@DOX NPs with or without laser irradiation. (G) Time-based absorption intensity reduction of DPBF. (H) Degradation of citraconic amide of P(CitAPDMAEMA)@Au@TiO_2_@DOX NPs under different pH. (n = 6, mean ± s.d.).

Another important property of P(CitAPDMAEMA)@Au@TiO_2_@DOX NPs was their adjustable photothermal effects. Τhe temperature changes in P(CitAPDMAEMA)@Au@TiO_2_@DOX NPs aqueous solutions with different concentrations under 635 nm laser irradiation at 2.0 W/cm^2^ were initially investigated (Figure 3A-B and Figure S9). The initial temperature of water and P(CitAPDMAEMA)@Au@TiO_2_@DOX solution is 25 °C. The solution temperature was able to be increased precisely by 32.5 °C (200 μg/mL). On the contrary, the temperature of pure water only increased by 2.8 °C. Therefore, cancer cells incubated with P(CitAPDMAEMA)@Au@TiO_2_@DOX (200 μg/mL) could be easily heated to temperatures higher than 50 °C within 5 min under laser irradiation at 2.0 W/cm^2^, thereby efficiently eliminating the cancer cells 40, 41. In order to further study the photothermal conversion efficiency of P(CitAPDMAEMA)@Au@TiO_2_@DOX, the temperature changes of the solution (200 μg/mL, 1.0 mL) under continuous 635 nm laser irradiation (2.0 W/cm^2^) as a function of time were recorded until the solution reached a stable temperature (Figure 3C). According to the results, the photothermal conversion efficiency could reach 20.4% (Figure 3D).

### Photodynamic Properties of P(CitAPDMAEMA)@Au@TiO_2_@DOX NPs

A proposed principle for the improved photodynamic performance of Au@TiO_2_ core-shell NPs was described in Figure 3E. The LSPR effect of AuNPs could extend the optical absorption into the red region 42. Therefore, upon red-light excitation, photo-electrons could be produced and transferred from the AuNPs to the conduction band of TiO_2_, leaving holes in the AuNPs. The reactive electrons combined with oxygen to form superoxide radicals (·O_2_^-^) and the holes could react with H_2_O to release hydroxyl radicals (·OH), which played important roles in PDT. Meanwhile, H_2_O_2_ could be easily adsorbed on the TiO_2_ surface and gave rise to surface complexes, which extended the photoresponse of TiO_2_ to the red region. The production of ·OH radicals showed the appearance of an electron-transfer process between the surface complexes and the TiO_2_ conduction band under red-light irradiation 35, 43. Consequently, the proposed reaction mechanism of H_2_O_2_ and TiO_2_ was as follows (Figure 3E): the H_2_O_2_ molecule was adsorbed onto the TiO_2_ surface and formed surface titanium-(IV) hydrogen peroxide complexes (Ti^IV^-OOH), which extended the photoresponse to the visible light region and could be activated by visible light. The active surface complex (Ti^IV^-OOH)* injected an electron to the conduction band of the Au@TiO_2_ core-shell NPs and generated e^-^ and Ti^IV^-·OOH, which might further give rise to Ti^IV^-OH and 1/2O_2_ 44. Therefore, the elevated concentration of O_2_ was helpful to yield ·O_2_^-^, which further enhanced the efficacy of PDT. Moreover, the conduction band electron further reacted with the adsorbed H_2_O_2_ and released the ·OH radical on the TiO_2_ surface. These highly reactive groups could be helpful to generate ROS on the surface of TiO_2_ and Au. It should be noted that in the core-shell NPs, most of the AuNPs surface was exposed to the medium. Therefore, both TiO_2_ and AuNPs could provide plenty of active sites to form ROS.

In addition, to explore the performance of P(CitAPDMAEMA)@Au@TiO_2_@DOX NPs in PDT, the ROS production capability of P(CitAPDMAEMA)@Au@TiO_2_@DOX NPs were investigated. Electron spin resonance (ESR) spectroscopy was applied to detect ·OH and ·O_2_^_^ generation by using 5,5'-dimethylpyrroline-1-oxide (DMPO) as a spin trap agent. According to the ESR spectrum, P(CitAPDMAEMA)@Au@TiO_2_@DOX NPs proved the characteristics of DMPO/·OH (Figure 3F) and DMPO/·OOH adduct (Figure S10A), which suggested the release of ·OH and ·O_2_^-^ and agreed with the literature 44. As a comparison, no ·OH and ·O_2_^-^ generation was detected in only DMPO or P(CitAPDMAEMA)@Au@TiO_2_@DOX NPs without laser irradiation. Interestingly, the intensity of the signal increased sharply upon 635 nm laser irradiation, confirming that P(CitAPDMAEMA)@Au@TiO_2_@DOX NPs could efficiently support the conversion of laser energy to chemical energy and release ROS for PDT. In order to quantify ROS generation, a photochemical method using 1,3-diphenylisobenzofuran (DPBF) as ROS probe in acetonitrile was employed 45. In the presence of P(CitAPDMAEMA)@Au@TiO_2_@DOX NPs, an obvious reduction in the absorption concentration of DPBF as a function of the irradiation time under 635 nm laser was observed. While in the control group, a slight decrease was shown (Figure 3G) when treated only with 635 nm laser, indicating the ability of P(CitAPDMAEMA)@Au@TiO_2_@DOX NPs to generate ROS under red-light irradiation. Furthermore, the ROS probe 2,7-dichlorodihydrofluorescein diacetate (H2DCFHDA) could generate green fluorescence after being oxidized by ROS, which was used to further prove intracellular ROS production under 635 nm laser irradiation. As shown in Figure S10B, a very weak fluorescence signal was presented for the resistant HeLa incubated with P(CitAPDMAEMA)@Au@TiO_2_@DOX NPs without irradiation. In contrast, the cells after irradiated with 635 nm laser exhibited a strong green fluorescence, demonstrating the intracellular production of ROS. As a result, P(CitAPDMAEMA)@Au@TiO_2_@DOX NPs could work as a new multifunctional theranostic nanoplatform for PTT and PDT when irradiated by a single 635 nm laser.

### Hydrolysis of Citraconic Amide of P(CitAPDMAEMA)@Au@TiO_2_@DOX NPs

Amides are acid labile when they have β-carboxylic acid groups, and primary amines can be exposed during their degradation. P(CitAPDMAEMA)@Au@TiO_2_ NPs was thought to experience conversion from zwitterionic to cationic P(CitAPDMAEMA)@Au@TiO_2_@DOX NPs due to citraconic amide degradation in acidic conditions. The process gave P(CitAPDMAEMA)@Au@TiO_2_@DOX NPs effective cancer cell targeting ability in acidic conditions. An amine-reactive fluorescence dye, fluorescamine, which reacts with the primary amine to generate fluorescent products 29. As such, with the fluorescamine method, the pH-dependent decomposition rate of the citraconic amide of P(CitAPDMAEMA)@Au@TiO_2_@DOX NPs could be detected by comparing the fluorescence before and after incubation. According to the results demonstrated in Figure 3H, about 97.2% of polymers retained the zwitterionic property in the physiological environment (pH 7.4), but they exposed the primary amine and quaternary ammonium under the acidic conditions, due to the removal of the -COO^-^ group in citraconic acid. The surface zeta potential of P(CitAPDMAEMA)@Au@TiO_2_@DOX NPs also changed after P(CitAPDMAEMA) hydrolysis (Figure S11).

### Hemolytic Properties of P(CitAPDMAEMA)@Au@TiO_2_@DOX NPs

**Figure 4:**
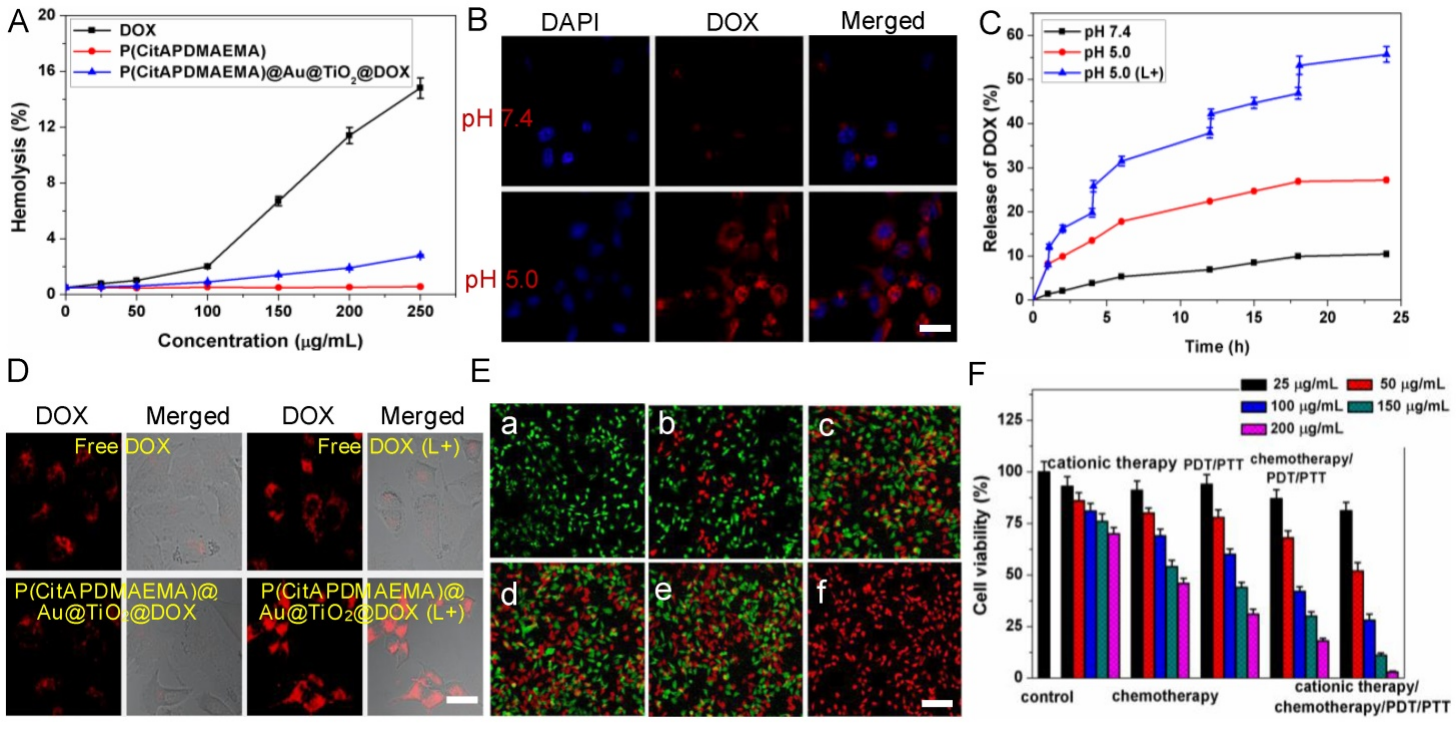
(A) Hemolytic rates of the DOX, P(CitAPDMAEMA) and P(CitAPDMAEMA)@Au@TiO_2_@DOX NPs. (n = 3, mean ± s.d.) (B) pH-dependent interaction between P(CitAPDMAEMA)@Au@TiO_2_@DOX NPs and HeLa cells. Scale bar: 30 μm (C) Drug release profile of P(CitAPDMAEMA)@Au@TiO_2_@DOX NPs at different conditions (pH 7.5, pH 5.5, and pH 5.5 +laser irradiation) within 24 h. (n = 3, mean ± s.d.) (D) CLSM images of HeLa cells incubated with free DOX and P(CitAPDMAEMA)@Au@TiO_2_@DOX NPs with or without laser irradiation (L+). Scale bar: 25 μm. (E) Fluorescence images of calcein AM/PI-stained HeLa cells incubated with various media: a) laser only; b) P(CitAPDMAEMA) (pH 5.0, cationic therapy); c) P(CitAPDMAEMA)@Au@TiO_2_@DOX NPs (pH 5.0, cationic therapy/chemotherapy); d) Au@TiO_2_ core-shell NPs (laser irradiation, PTT/PDT; e) Au@TiO_2_@DOX NPs (laser irradiation, chemotherapy/PTT/PDT); f) P(CitAPDMAEMA)@Au@TiO_2_@DOX NPs (ationic therapy /chemotherapy/ PTT/PDT). Scale bar: 100 μm (F) Cytotoxicity of HeLa cells after different treatments. (n = 6, mean ± s.d.).

For potential clinical applications, anticancer agents should have no detrimental effects on the human body. To determine the cytotoxicity of anticancer agents that are particularly relevant to the intravenous application, hemolysis is a standard method 46. Fresh whole rabbit blood was applied to measure the hemotoxicity of P(CitAPDMAEMA), DOX, and P(CitAPDMAEMA)@Au@TiO_2_@DOX NPs (Figure 4A). Among the tested concentrations, few red blood cells (RBCs) were lysed, indicating a very low hemolytic activity of P(CitAPDMAEMA). Meanwhile, no detectable hemolytic activity of P(CitAPDMAEMA)@Au@TiO_2_ NPs on RBCs during the entire experimental DOX concentration range was found. It should be noted that DOX concentration could decide the hemolytic activity of soluble DOX. Hemolysis was not measured when the tested concentration of DOX below 100 μg/mL, while at 250 μg/mL concentration, 14.3% hemolysis was detected. These results indicated that the P(CitAPDMAEMA)@Au@TiO_2_@DOX NPs were safe for blood-contact usage and held potential for intravenous administration.

### pH-dependent Binding of P(CitAPDMAEMA)@Au@TiO_2_@DOX NPs to HeLa cells

The zwitterionic biocompatible P(CitAPDMAEMA)@Au@TiO_2_@DOX NPs were thought to change into cationic and bind to the surface of cancer cells in the acidic condition. The binding of P(CitAPDMAEMA)@Au@TiO_2_@DOX NPs to cancer cells under acidic condition was evaluated by confocal laser scanning microscopy (CLSM), and HeLa cells were applied as the model cancer strain. P(CitAPDMAEMA)@Au@TiO_2_@DOX NPs were firstly labeled with DOX and then incubated with HeLa cells in PBS with different pH for 1 h. From Figure 4B, we could see the strong fluorescence in a group of HeLa cells at pH 5.0, while a low fluorescent signal was detected in the group at pH 7.4.

### DOX Releasing

In order to evaluate the properties of P(CitAPDMAEMA) as drug release tools, the *in vitro* release of DOX from the P(CitAPDMAEMA)@Au@TiO_2_@DOX NPs was monitored by applying the dialysis membrane against PBS. In order to investigate the pH-dependent drug release, the released DOX was collected by dialyzing P(CitAPDMAEMA)@Au@TiO_2_@DOX NPs in PBS with different pHs. After 24 h, about 27.2% of DOX was released from the nanoparticles in pH 5.0, in contrast to 10.4% of DOX that was released in pH 7.4 (Figure 4C). Next, it was investigated whether red light-induced photothermal heating could also induce DOX release. P(CitAPDMAEMA)@Au@TiO_2_@DOX NPs in PBS (pH 5.0 or 7.4) were irradiated by 635 nm laser at 2.0 W/cm^2^ for 5 min at different time points. As compared to the DOX amount released in dark without laser irradiation, red light-stimulus dramatically increased the DOX release. Besides, with the same laser irradiation, the DOX release seemed to be more apparent in the lower pH environment compared to that in the physiological pH condition. These results indicated that the P(CitAPDMAEMA) molecules could be effectively applied as tools to instantly turn on/off the drug release, controlled by an environmental pH and red light.

In addition, it was examined whether the red-light induced drug release action of P(CitAPDMAEMA)@Au@TiO_2_@DOX NPs also exists inside cells 47. Due to the fluorescence quenching of DOX after loading on the nano-carriers, reoccurrence of the DOX fluorescence could become an indicator for drug release. As shown in the CLSM images (Figure 4D), no dramatic discrepancy was detected in free DOX-treated cells after laser irradiation, and the drug molecules were evenly dispersed in the cytoplasm and nuclei. By comparison, obvious improved DOX fluorescence was detected in cells incubated with P(CitAPDMAEMA)@Au@TiO_2_@DOX NPs after laser irradiation, suggesting DOX was release from the nano-carriers. Analysis by ImageJ software on LSCM images disclosed the quantitative evidence that NIR laser irradiation promoted drug release from P(CitAPDMAEMA)@Au@TiO_2_@DOX NPs in cells (Figure S12A). These results collectively proved that red-light irradiation could effectively trigger intracellular drug release from P(CitAPDMAEMA)@Au@TiO_2_@DOX NPs.

### Cell Toxicity

The *in vitro* cytotoxicity of as-prepared P(CitAPDMAEMA) and Au@TiO_2_ core-shell NPs were then evaluated with HeLa cells as a model by 3-(4,5-dimethyl-2-thiazolyl)-2,5-diphenyl-2-H-tetrazolium bromide (MTT) assay (Figure S12B). No obvious cytotoxicity to the P(CitAPDMAEMA) or Au@TiO_2_ core-shell NPs-transfected HeLa cells was observed as the P(CitAPDMAEMA) and Au@TiO_2_ core-shell NPs concentration ranged from 50 to 150 μg/mL, while a slight reduction of cell viability appeared at extremely high concentrations (200 μg/mL), indicating their good biocompatibility.

Subsequently, the efficacy of the cationic therapy/chemotherapy/PDT/PTT and the cytotoxicity of the produced P(CitAPDMAEMA)@Au@TiO_2_@DOX NPs were investigated* in vitro*. Normal and dead cells were determined by applying calcein AM (green) and PI (red) staining, respectively. In the laser-only (2.0 W/cm^2^) groups, no dead cells was observed, due to green fluorescence displayed by all cells (Figure 4E,a). This phenomenon showed the inability of the heat generated from pure water under irradiation to cause cancer cell death. Since P(CitAPDMAEMA) is able to bind to HeLa cells and can be converted to cationic P(CitAPDMAEMA) in the acidic environment, it was thought to obtain anticancer efficacy. In Figure S13, it was observed that P(CitAPDMAEMA) pretreated at pH 7.4, but did not show any effect on the growth of the HeLa cells, which was due to its zwitterionic nature at neutral pH. However, in the case of P(CitAPDMAEMA) pretreated at pH 5.0 (Figure 4E,b), the growth of the HeLa cells was obviously restrained. This result could be explained that P(CitAPDMAEMA) effectively absorbed the negative charge on the membrane of HeLa cells in the acidic environment, which inhibited the growth and metastasis of HeLa cells. Meanwhile, some cells got damage after incubating with 200 μg/mL Au@TiO_2_@DOX NPs (Figure 4E,c) because of the release of DOX. After laser irradiation at 2.0 W/cm^2^ and incubation with 200 μg/mL Au@TiO_2_ core-shell NPs (Figure 4E,d) and Au@TiO_2_@DOX NPs (Figure 4E,e), most of the cells were destroyed. On the contrary, after laser irradiation at 2.0 W/cm^2^ and incubation with 200 μg/mL P(CitAPDMAEMA)@Au@TiO_2_@DOX NPs (Figure 4E,f), almost all cells were killed, as demonstrated by the obtained intense homogeneous red fluorescence. The result was attributed to the fact that P(CitAPDMAEMA)@Au@TiO_2_@DOX NPs were able to simultaneously release DOX at high power doses, converting back to cationic P(CitAPDMAEMA), and generate ·OH/·O_2_^-^, and produce heat to efficiently kill cells by combined cationic therapy/ chemotherapy/PDT/PTT using a single red laser. Moreover, the cationic therapy/chemotherapy/PDT/ PTT efficacy of the P(CitAPDMAEMA)@Au@TiO_2_@DOX NPs was quantitatively assessed by the MTT assay. Among cationic therapy, chemotherapy, PDT/PTT, and chemotherapy/PDT/PTT groups, cell viability decreased with the increasing dose of the sample, while some cells remained alive even at a high sample concentration (200 μg/mL). However, in the cationic therapy/chemotherapy/PDT/PTT group, a mortality rate of ~98% was obtained, indicating that the simultaneous effects of cationic therapy/chemotherapy/PDT/PTT were able to effectively kill cancer cells (Figure 4F). These results agreed with the calcein AM and PI staining findings.

### MR Imaging

**Figure 5:**
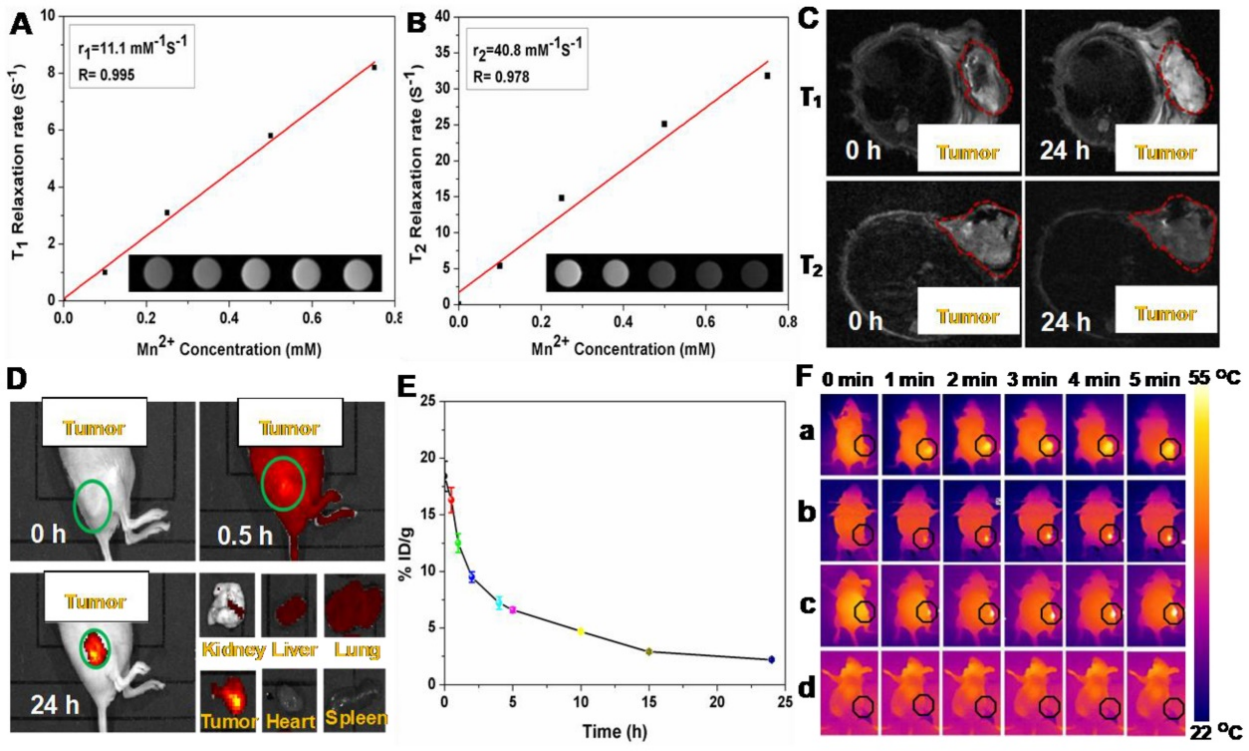
The (A) *T*_1_/(B) *T*_2_ relaxation rates and *T*_1_/*T*_2_-MR images (insets) of n@P(CitAPDMAEMA)@Au@TiO_2_@DOX NPs solutions with different Mn^2+^ concentrations. (C) *In vivo T*_1_/*T*_2_-MR images of HeLa-tumor-bearing mice taken 0 h and 24 h post i.v. injection with Mn@P(CitAPDMAEMA)@Au@TiO_2_@DOX NPs. (D) The fluorescence images of HeLa-tumor-bearing mice after i.v. injected with P(CitAPDMAEMA)@Au@TiO_2_@DOX NPs. The tissues were exploited for ex *vivo* fluorescence images after 24 h i.v. injection. (E) Blood circulation of Mn@P(CitAPDMAEMA)@Au@TiO_2_@DOX NPs after i.v. injection (n = 5, mean ± s.d.). (F) IR thermal images of HeLa-tumor-bearing mice injected with (a) Au@TiO_2_ core-shell NPs, (b) Au@TiO_2_@DOX NPs, (c) P(CitAPDMAEMA)@Au@TiO_2_@DOX NPs, and (d) PBS under 635 nm laser irradiation (2.0 W/cm^2^, 5 min).

It is known that the carboxyl and hydroxyl groups can easily chelate different kinds of metal ions, such as Cu^2+^, Mn^2+^, and Fe^3+^ 47, 48. Among these metal ions, Mn^2+^ has been widely utilizing as a contrast agent for MRI. Based on the present system, Mn^2+^ was successfully loaded on P(CitAPDMAEMA)@Au@TiO_2_@DOX NPs by intrinsically chelating with carboxyl and hydroxyl groups on the surface of NPs. Interestingly, the obtained Mn@P(CitAPDMAEMA)@Au@TiO_2_@DOX NPs demonstrated a significant intensity-dependent brightening function under *T*_1_-weighted MRI (inset of Figure 5A), as well as a darkening function under *T*_2_-weighted MRI (inset of Figure 5B). The r_1_ and r_2_ relaxivities of the Mn@P(CitAPDMAEMA)@Au@TiO_2_@DOX NPs were detected at 11.1 mM^-1^s^-1^ and 40.8 mM^-1^s^-1^, respectively.

Therefore, the developed Mn@P(CitAPDMAEMA)@Au@TiO_2_@DOX NPs could be applied as a contrast agent for both *T*_1_-weighted and *T*_2_-weighted MRI. Furthermore, *in vivo* MRI was applied on mice. Mice bearing HeLa tumors were initially intravenously (i.v.) injected with Mn@P(CitAPDMAEMA)@Au@TiO_2_@DOX NPs and then imaged on a 3.0 T MRI system. Significant brightening or darkening effects were observed in the tumor region of mice compared with the pre-injected image (Figure 5C), suggesting high tumor accumulation of those NPs after systemic administration. Furthermore, the stronger *T*_1_- and *T*_2_-weighted MRI signal could be observed in the tumor region at 24 h post-injection. ImageJ was used to measure the region-of-interest (ROI) quantification (Figure S14) which further confirmed the enhancement of signal intensity in the tumor region after i.v. injection of Mn@P(CitAPDMAEMA)@Au@TiO_2_@DOX NPs. These results indicated that the effective tumor accumulation of Mn@P(CitAPDMAEMA)@Au@TiO_2_@DOX NPs via EPR effect and blood circulation.

### Fluorescent Imaging

The whole-body fluorescence imaging was used to detect the biodistribution of DOX in HeLa tumor-bearing mice at specific time points following intravenous injection of P(CitAPDMAEMA)@Au@TiO_2_@DOX NPs (5 mg/kg). As shown in Figure 5D, P(CitAPDMAEMA)@Au@TiO_2_@DOX NPs could spread into the body of mice. Interestingly, the drug delivery nanosystem based on the P(CitAPDMAEMA)@Au@TiO_2_@DOX NPs was accumulated in the tumor at 24 h. This result was validated by the quantitative detection of DOX intensity in the normal tissue and the tumor regions (Figure S15). It was speculated that P(CitAPDMAEMA)@Au@TiO_2_ NPs were captured by the liver, spleen, and lung tissues, which prolonged the blood-circulation lifetime. As a result, this progress increased the efficiency of the P(CitAPDMAEMA)@Au@TiO_2_@DOX NPs to recognize and gather at the tumor site 49. In particular, the gathering of P(CitAPDMAEMA)@Au@TiO_2_@DOX NPs in the heart decreased, indicating that the nanosystem could decrease cardiac muscle toxicity caused by DOX. These findings showed that P(CitAPDMAEMA)@Au@TiO_2_@DOX NPs were able to recognize the tumor, accumulate in the tumor, and decrease the side effects of DOX.

### Blood Circulation and Biodistribution

Prior to combination therapy, the *in vivo* behavior of the P(CitAPDMAEMA)@Au@TiO_2_@DOX NPs were examined. Female Balb/c mice were i.v. injected with P(CitAPDMAEMA)@Au@TiO_2_@DOX NPs (5 mg/kg, 200 μL). Blood was collected from the mice at every time point. After being dispersed in lysis buffer and extracted with HCl/isopropanol, DOX was measured by fluorescence in order to assess its intensity in the blood 47. As seen in Figure 5E, the signals of DOX gradually decreased over time following a two-compartment model, where the first (t_1/2_(α)) and second (t_1/2_(β)) phases of circulation half-lives were calculated to be 1.92 ± 0.24 h and 9.86 ± 0.52 h, respectively. Furthermore, half of P(CitAPDMAEMA)@Au@TiO_2_@DOX NPs were rapidly excreted through the urine and feces route 24 h after injection due to their nanoscale (Figure S16). After 24 h intravenous injection, a large part of P(CitAPDMAEMA)@Au@TiO_2_@DOX NPs appeared in the liver and kidney because of the reticuloendothelial system clearance. Around 23.7% of the P(CitAPDMAEMA)@Au@TiO_2_@DOX NPs were accumulated into the tumor through the EPR effect and the cationic targeting effect (Figure S17). It could also modify the targeted ligands to improve its accumulation efficiency. P(CitAPDMAEMA)@Au@TiO_2_@DOX NPs could be not only passively target the tumor tissue, but also evaluate the tumor site/dimension/morphology by fluorescent/infrared thermal/MR treble-modal imaging which was able to guide the simultaneous PTT and PDT.

### *In Vivo* Photothermal Imaging

In the light of good photothermal conversion of P(CitAPDMAEMA)@Au@TiO_2_@DOX NPs, we evaluated *in vivo* photothermal efficiency in the tumor model. Female Balb/c mice were i.v. injected with Au@TiO_2_ core-shell NPs, Au@TiO_2_@DOX NPs, P(CitAPDMAEMA)@Au@TiO_2_@DOX NPs, and PBS. After 6 h, tumor regions of mice were irradiated by 635 nm laser with a power density of 2.0 W/cm^2^ for 5 min. The change of temperature during irradiation was detected by an IR thermal camera (Figure 5F and Figure S18). The temperatures of tumor in the mice injected with Au@TiO_2_@DOX NPs, Au@TiO_2_ core-shell NPs, and P(CitAPDMAEMA)@Au@TiO_2_@DOX NPs demonstrated a rapid increase and then maintained at 54 °C during 635 nm laser irradiation. In contrast, little change was measured in the tumor temperature of the mice injected with P(CitAPDMAEMA)@Au@TiO_2_ NPs and PBS under 635 nm laser irradiation using the same parameters.

### *In Vivo C*ombined Therapy

**Figure 6:**
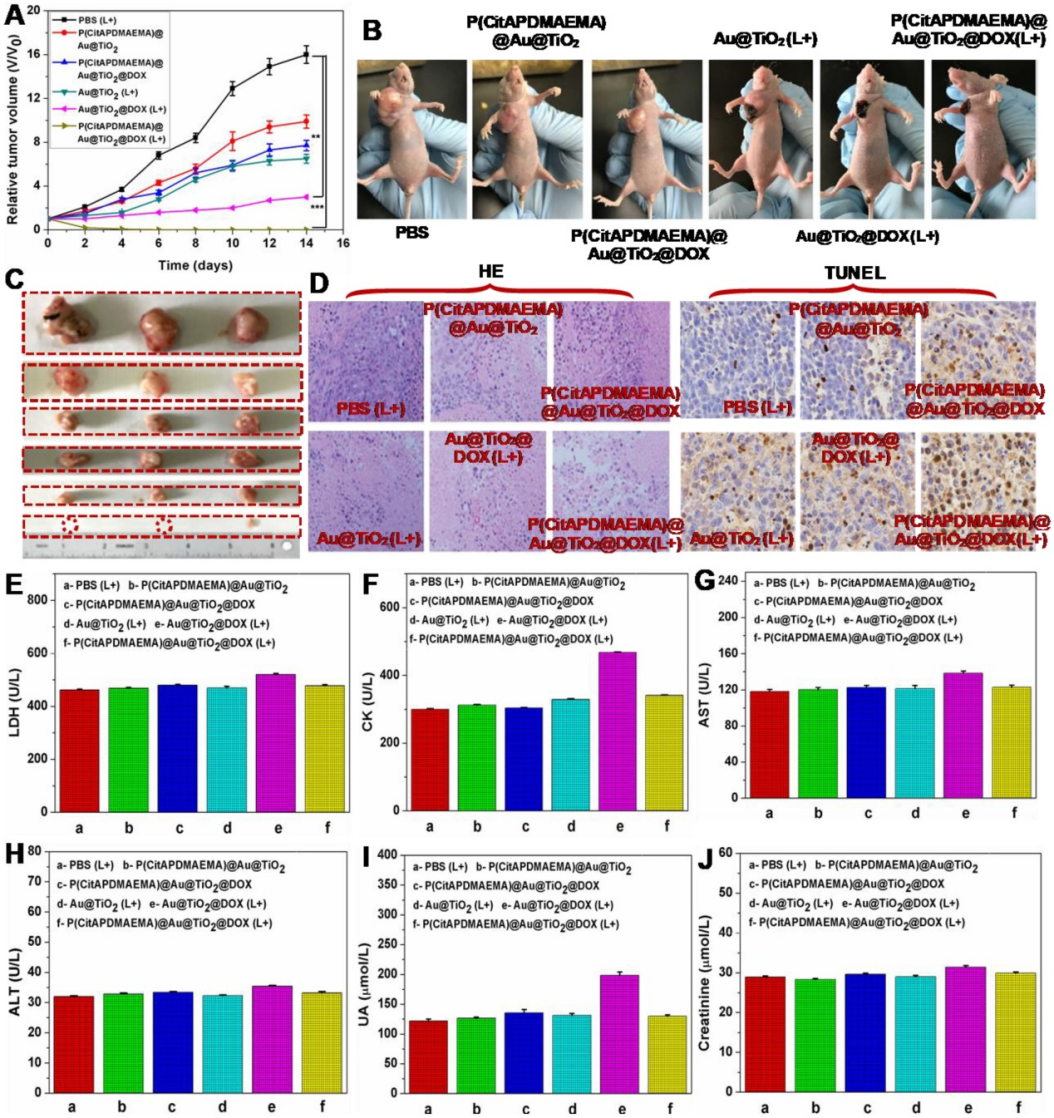
(A) Tumor growth curves for mice with various treatment. (n = 5, mean ± s.d., **P<0.01, and ***P<0.001). (B) The digital photographs of mice 14th days with various treatments. (C) Representative photographs of the excised tumors 14th days with various treatments. (up to bottom: PBS (L+), P(CitAPDMAEMA)@Au@TiO_2_ NPs, P(CitAPDMAEMA)@Au@TiO_2_@DOX NPs, Au@TiO_2_ core-shell NPs (L+), Au@TiO_2_@DOX NPs (L+), and P(CitAPDMAEMA)@Au@TiO_2_@DOX NPs (L+)). (D) TUNEL and HE staining of tumor slices collected from different mice groups after 14 days treatment. (E-J) Blood biochemistry analysis (LDH, CK, AST, ALT, UA, and Creatinine) of the different mice groups after 14 days treatment. (n = 5, mean ± s.d.).

Based on the mentioned *in vivo* imaging results, *in vivo* cancer treatment with P(CitAPDMAEMA)@Au@TiO_2_@DOX NPs was carried out (Scheme 1). Female Balb/c mice were subcutaneously injected into their backs with 1×10^6^ HeLa cells. After one week, the tumors grew to 60 mm^3^ and the mice were divided into 6 groups: P(CitAPDMAEMA)@Au@TiO_2_ NPs (1), P(CitAPDMAEMA)@Au@TiO_2_@DOX NPs (2), PBS (plus Laser) (3), Au@TiO_2_@DOX NPs (plus Laser) (4), Au@TiO_2_ core-shell NPs (plus Laser) (5), and P(CitAPDMAEMA)@Au@TiO_2_@DOX NPs (plus Laser) (6). The drug and drug-loaded NPs were i.v. injected into each mouse through the tail vein. After the treatment by laser, the tumor volume was measured by a digital caliper every two days for a total of two weeks (Figure 6A-B). The combination therapy group of the P(CitAPDMAEMA)@Au@TiO_2_@DOX NPs with laser irradiation, demonstrated an obvious inhibition of tumor growth. The Au@TiO_2_ core-shell NPs and Au@TiO_2_@DOX NPs with laser irradiation presented a small inhibitory function during the early days, but grew normally later. The P(CitAPDMAEMA)@Au@TiO_2_ NPs and P(CitAPDMAEMA)@Au@TiO_2_@DOX NPs treatment groups seemed to have a minimal influence on tumor growth. These results were also confirmed by representative tumor images (Figure 6C) at day 14. Slices of tumors stained with hematoxylin and eosin (HE) and TUNEL staining (Figure 6D) were obtained, in order to further assess the therapeutic function of the various treatments. It was shown that the combination treatment group P(CitAPDMAEMA)@Au@TiO_2_@DOX NPs with laser irradiation showed the most effective damage on most of the tumor cells, while the other five groups depicted little or no damage on tumor cells, which had normal membrane morphology and nuclear structures.

In order to investigate whether these treatments would induce toxicity, the body weight of the mice was monitored (Figure S19) and histology of organ samples including lung, liver, spleen, kidney, and heart was performed (Figure S20). The body weight of the mice in the treated groups was about the same as those in the control group. No loss of body weight or other serious toxic effects were observed. Furthermore, the histological analysis did not reveal any serious irreversible pathological alterations or injuries in the organs of the mice of all groups. Moreover, the toxicity of the P(CitAPDMAEMA)@Au@TiO_2_@DOX NPs with laser irradiation was detected by blood biochemical analysis. As can be seen in Figure 6E-F, the P(CitAPDMAEMA)@Au@TiO_2_@DOX NPs with laser irradiation group demonstrated no apparent increase in lactate dehydrogenase (LDH) and creatine kinase (CK) levels, which were necessary parameters for heart function. In addition, the analysis of liver function (Figure 6G-H) [aspartate aminotransferase (AST) and alanine aminotransferase (ALT)] and kidney function (Figure 6J-I) [uric acid (UA) and creatinine] parameters did not show any damage to liver and kidney in the mice treated with P(CitAPDMAEMA)@Au@TiO_2_@DOX NPs with laser irradiation. This phenomenon suggested that the P(CitAPDMAEMA)@Au@TiO_2_@DOX NPs combination treatment did not induce any toxicity. All results above clearly showed that the P(CitAPDMAEMA)@Au@TiO_2_@DOX NPs could serve as powerful *in vivo* anticancer agents.

## Conclusions

In conclusion, we have successfully designed and constructed a multifunctional nanohybrid for imaging-guided combination treatment of cancer. The resultant P(CitAPDMAEMA)@Au@TiO_2_@DOX NPs possessed the following features: (1) rapid binding to cancer cells under acidic conditions; (2) high photothermal conversion efficiency under 635 nm laser irradiation; (3) efficient ROS release upon 635 nm laser irradiation; (4) induced DOX release by external red-light and at intracellular pH values. In addition, those NPs possessed obvious contrasts in MRI after the chelation with Mn^2+^. An imaging-guided cancer therapy, which combined with those functional parts, was shown in mouse tumor model experiments. The cationic therapy/ chemotherapy/PTT/PDT delivered by the P(CitAPDMAEMA)@Au@TiO_2_@DOX NPs showed a significant combined effect in destructing tumors using. Moreover, the tiny size of the nanoparticles allowed for partial renal clearance and high tumor uptake *in vivo*, making the theranostic agent a promising candidate for future clinical translation.

## Experimental Section

### Chemicals

2-(Dimethylamino)ethyl methacrylate (DMAEMA, 98%), citraconic anhydride (Cit, 98%), 3-(4,5-dimethylthiazol-2-yl)-2,5-diphenyltetrazolium bromide (MTT, 98%) and 4-cyanopentanoic acid dithiobenzoate (CPADB, 98%) were purchased from Sinopharm Chemical Reagent CO, Ltd., China. Doxorubicin (DOX) were purchased from Tianjin Biolite Biotech Co., Ltd. The dialysis tube membrane with molecular weight cutoff (MWCO) of 1000 was purchased from Peg Bio, Suzhou, China. All aqueous solution used in experiment was deionized water (DI, 18.2 MΩ·cm) obtained from Milli-Q water purification system.

### Instruments and characterization

The infrared spectrum was performed on a Nexus 670 FTIR type (Nicolet). The X-ray diffraction (XRD) analysis was performed using a D/Max 2500V/PC diffractometer (Rigaku Corporation, Japan). UV-Vis spectroscopy measurements were performed on a Cary 5000 UV-Vis-NIR spectrometer (Varian). The surface composition and element analysis of the samples were recorded using X-ray photoelectron spectroscopy (XPS, EscaLab-250, Thermo, USA). The hydrodynamic size and zeta-potential were measured on a Malvern ZEN 3600 Zetasizer (Malvern Instruments, UK). The transmission electron microscopy (TEM) images were acquired on a JEM-2100F TEM. The fluorescent images of cells were acquired by Confocal Scanning Laser Microscope (CSLM, TI-E-A1R, Nikon, Japan). The infrared thermal images were recorded with a PTT monitoring system MG33 (Shanghai Magnity Electronics Co. Ltd.). The methods used for material characterization are displayed in the experimental section of the supporting information.

## Supplementary Material

Supplementary experimental section, schemes and figures.
