# Supplementary material for: Zwitterionic Polymer-Gated Au@TiO2 Core-Shell Nanoparticles for Imaging-Guided Combined Cancer Therapy
Source: Theranostics. 2019 Jul 9;9(17):5035–48. doi: 10.7150/thno.35418 (PMC6691384; doi:10.7150/thno.35418)
Supplement: Supplementary file 1 — Supplementary experimental section, schemes and figures. [file thnov09p5035s1.pdf]

Supporting Information

pH-Sensitive Zwitterionic Polymer-Gated Au@TiO_2 Core-Shell Nanoparticles for Imaging-guided Cancer Synergistic Therapy

Tao Zheng[1], Wentao Wang[2], Fan Wu[2], Ming Zhang[1,2*], Jian Shen[2*], Yi Sun[1*]

[1]Department of Health Technology, Technical University of Denmark, Kongens Lyngby, 2800, Denmark;

[2]Jiangsu Collaborative Innovation Center for Biomedical Functional Materials, School of Chemistry and Materials Science, Nanjing Normal University, Nanjing 210023, P. R. China

Corresponding author: Ming Zhang, mzhan@dtu.dk; Jian Shen, jshen@njnu.edu.cn; Yi Sun, suyi@dtu.dk

Experiment Section

Synthesis of $Au@TiO_2$ core-shell NPs. AuNPs were synthesized according to previous a AuNPs method [1], as shown in TEM image (Figure S1A). The as-prepared AuNPs solution (8 mL) was sequentially mixed with TiF_4 (40 mM, 0.5 mL) and water (8 mL), and the reaction mixture was stirred for 5 min. Subsequently, ethanol (14 mL) was added, and the resulting mixture was stirred for 10 min. Finally, the mixture was transferred to Teflon-lined stainless steel autoclaves and incubated at 180 °C for 6 h. To isolate the $Au@TiO_2$ core-shell NPs, the reaction mixture was centrifuged at 6000 rpm for 8 min. Then, the concentrated products were dried at 60 °C for 6 h in an electric oven.

Drug loading. $Au@TiO_2$ core-shell NPs (1 mg) was firstly dissolved 10 mL PBS solution (pH 7.4) containing 2.0 mg DOX under magnetic stirring at 37 °C for 48 h. The mixture was then separated with an external magnetic field. In order to remove unloaded DOX adsorbed physically on the outer surface of the carbon shell of $Au@TiO_2$ core-shell NPs, the precipitate was redispersed in 10 mL PBS solution (pH 7.4) and further purified by repeated separation and washing until the separated solution became clear. All the washed and separated solutions were collected and combined. The amount of unloaded free-DOX molecules in the combined solution was quantified by a UV-vis spectrophotometer at 480 nm. The drug loading content of $Au@TiO_2$ core-shell NPs was detected by UV-Vis-NIR spectra.

Synthesis of P(CitAPDMAEMA)@$Au@TiO_2$@DOX. The synthetic procedure was the same as the previously reported method by Liu and co-workers [2]. To perform P(CitAPDMAEMA) coating, 2 mg of $Au@TiO_2$@DOX NPs was dispersed in 1 mL of P(CitAPDMAEMA) aqueous solution (0.4 mg/mL, pH 7.5). After the mixture was sonicated for 10 min and stirred for 30 min, the P(CitAPDMAEMA)-coated $Au@TiO_2$@DOX NPs were centrifuged and washed with H_2O to remove excessive P(CitAPDMAEMA). The product was obtained by freeze-drying. The obtained $Au@TiO_2$@DOX NPs conjugated with $Au@TiO_2$@DOX NPs on is referred to as

P(CitAPDMAEMA)@Au@TiO_2@DOX NPs.

Preparation of Mn^{2+} chelated P(CitAPDMAEMA)@Au@TiO_2@DOX. The P(CitAPDMAEMA)@Au@TiO_2@DOX NPs (1.0 mg/mL) were labeled with Mn^{2+} by adding of $MnCl_2$ solution (1.0 mg/mL) at the neutral condition. After 4 h stirring at 37 ^oC, the samples were centrifuged for several times to remove unbound excess Mn^{2+}. The concentration of Mn^{2+} in the obtained nano-complexes was determined to be 78 mg/g by inductively coupled plasma-mass spectrometry (ICP-MS) analysis.

Photothermal performance of P(CitAPDMAEMA)@Au@TiO_2@DOX NPs. Measurement of photothermal performance: aqueous-dispersion P(CitAPDMAEMA)@Au@TiO_2@DOX NPs (1.0 mL) with different concentrations (0-200 μg/mL) were introduced in a quartz cuvette and irradiated with 635 nm laser at a power density of 2.0 W/cm^2 for 5 min to measure the photothermal conversion performance of P(CitAPDMAEMA)@Au@TiO_2@DOX NPs. P(CitAPDMAEMA)@Au@TiO_2@DOX NPs (200 μg/mL) were then irradiated by laser power densities (2.0 W/cm^2) to investigate the laser power dose effect. Temperature was recorded every 1 s by a digital thermometer using a thermocouple probe with an accuracy of 0.1 °C. Real-time thermal imaging of the samples was recorded using a thermal infrared camera and was quantified by examiner software.

Photothermal conversion efficiency was evaluated by recording the temperature change of the aqueous dispersion (200 μg/mL) as a function of time under 635 nm laser irradiation at a power density of 2.0 W/cm^2 until the solution reached a steady-state temperature. Photothermal conversion efficiency, η, was calculated using Equation (1), as described by referring to the previous literatures.

$$\eta=[\, hA(T_{max} - T_{surr}) - Q_{Dis}] \,/\, [\, I(1-10^{-A\lambda})] \qquad (1)$$

where h is the heat transfer coefficient, A is the surface area of the container, T_{max} is the equilibrium temperature, T_{surr} is the ambient temperature of the surroundings, Q_{Dis} expresses the heat dissipation from the light absorbed by the quartz sample cell, I is incident laser power (2.0 W/cm^2), and A_{635} is the absorbance of

P(CitAPDMAEMA)@Au@TiO_2@DOX NPs at 635 nm (0.09). The value of *hA* is derived according to Equation (2):

$$\tau_S = m_D C_D / hA \qquad (2)$$

where τ_s is the sample system time constant, m_D and C_D are the mass (1.0 g) and heat capacity (4.2 J/g) of deionized water used as a solvent, respectively. Q_{Dis} (210 mW) was independently measured using a quartz cuvette cell containing pure water without P(CitAPDMAEMA)@Au@TiO_2@DOX NPs.

In order to get the τ, we herein introduce a parameter θ, which is defined as the ratio of ΔT to ΔT_{max}

$$\theta = \Delta T / \Delta T_{max} \qquad (3)$$

ΔT is the temperature change, which is defined as $T\text{-}T_{surr}$ (T and T_{surr} are the solution temperature and ambient temperature, respectively), and ΔT_{max} is the temperature change at the maximum steady-state temperature

$$t = -\tau \ln(\theta) \qquad (4)$$

Thus, τ can be determined by applying the linear time data from the cooling period versus $\ln(\theta)$. Therefore, *hA* can be calculated.

Photodegradation of DPBF. Typically, 100 mL of ethanol solution with DPBF and P(CitAPDMAEMA)@Au@TiO_2@DOX NPs were stirred in the dark for 100 min to reach the adsorption/desorption equilibrium prior to the test. The mixture was irradiated by laser of 635 nm for 5 min. The samples were taken at different times for UV-vis measurements. For comparison, the ROS production of acetonitrile solution without P(CitAPDMAEMA)@Au@TiO_2@DOX NPs was also measured in parallel under the same condition.

Hydrolysis of citraconic amide of P(CitAPDMAEMA)@Au@TiO_2@DOX. P(CitAPDMAEMA)@Au@TiO_2@DOX NPs was dissolved in DI water to a final concentration of 200 μg/mL, and 50 μL of this stock solution was mixed with citric acid-PBS (450 μL). The same process was carried out in PBS (pH 6.0 or 5.0). These mixtures were incubated at 37 °C for 24 h, and at each predetermined time interval, 10 μL of each sample was taken out and diluted into 1 mL of borate buffer (pH 9.3).

These mixtures were added by 10 μL of fluorescamine solution in N,N-dimethylformamide and incubated at 25 °C for 10 min. The fluorescence in the combined solution was quantified by a fluorescence spectrophotometer under an excitation wavelength of 375 nm.

DOX release. NIR-simulated release of DOX was carried out by 635 nm laser irradiation. In a typical experiment, the P(CitAPDMAEMA)@Au@TiO_2@DOX NPs was dissolved in 5 mL PBS solution at different pH values (5.0 and 7.4). At different time points, the samples were irradiated by an 635 nm laser for 5 min with the power density of 2.0 W/cm^2. After treatment, 500 μL of solution was collected and centrifuged to obtain released DOX, which was measured and determined by UV-Vis spectrum.

To investigate the NIR-triggered intracellular drug release, HeLa cells were incubated with P(CitAPDMAEMA)@Au@TiO_2@DOX NPs or free DOX at the same concentration of DOX (15 μM) for 0.5 h, then treated. with or without irradiation by the 635 nm laser for 10 min under the power density about 2.0 W/cm^2. All cells were washed twice with PBS before CSLM imaging.

Hemolysis assay of P(CitAPDMAEMA)@Au@TiO_2@DOX NPs. Fresh rabbit whole blood was offered from Jiangsu Blood Center. Briefly, fresh rabbit whole blood was diluted in PBS to achieve 8% blood content. P(CitAPDMAEMA), P(CitAPDMAEMA)@Au@TiO_2@DOX NPs (W_{DOX} : $W_{Au@TiO2}$ = 3), and DOX solutions (100 μL) of different concentration were mixed with equal volume of the diluted blood suspension. The mixtures were incubated at 37 °C for 2 h, and then subjected to centrifugation at 1500 rpm for 10 min. After which the supernatant (100 μL) was transferred into a 96-well plate. The hemoglobin release was evaluated by measuring the optical absorbance at 576 nm using a microplate reader.

pH-Dependent P(CitAPDMAEMA)@Au@TiO_2@DOX/HeLa cells binding test. HeLa cells were purchased from Nanjing KeyGen Biotech Co.,Ltd. and cultured

under standard conditions (37 ºC, 5% CO_2). HeLa cells were seeded into each well of chambered coverglass at a density of 1.5×10^4 and incubated with after 24 h seeding. About P(CitAPDMAEMA)@Au@TiO_2@DOX NPs solution (250 µL, 100 µg/mL) in different pH buffers (pH 7.4 and 5.0) was mixed with equal volume of HeLa cells suspension (250 µL) in chambered coverglass. After 1 h incubation, the medium was removed and the cells were washed 3 times using PBS (pH 7.4). Fluorescence images were collected on a CSLM under 543-nm excitation immediately after PBS (pH 7.4) washing.

Cytotoxicity of P(CitAPDMAEMA) and Au@TiO_2 NPs. To determine the cytotoxicity of nanocomplexes, HeLa cells were seeded in 96-well plates with 1×10^4 cells/well. Then, different concentrations of P(CitAPDMAEMA) and Au@TiO_2 core-shell NPs were added and co-incubated with cells for 24 h. Afterwards, the cells were washed with free cell culture medium for twice. The relative cell viabilities compared with untreated groups were measured by the MTT assay.

***In vitro* combination therapy.** HeLa cells were incubated with and without P(CitAPDMAEMA)@Au@TiO_2@DOX NPs (W_{DOX}:$W_{Au@TiO2}$=3, 200 µg/mL) for 4 h and then irradiated by 635 nm laser at different power densities (2.0 W/cm^2) for 5 min. The cells were costained with calcein AM and propidium iodide for 30 min, washed with PBS, and then imaged by a CSLM. An MTT assay was carried out to determine cell viabilities under various conditions to further confirm the cytotoxicity and phototherapy efficacy of P(CitAPDMAEMA)@Au@TiO_2@DOX NPs (W_{DOX}:$W_{Au@TiO2}$=3). For zwitterionic therapy, HeLa cells were seeded into 96-well plates and incubated with different concentrations of P(CitAPDMAEMA) prepared using PBS (pH 7.4 and 5.0) at 37 °C for 24 h in a humidified 5% CO_2 atmosphere, each concentration of P(CitAPDMAEMA) was carried out with six parallel groups. For *in vitro* chemothearpy, HeLa cells were seeded into 96-well plates and incubated with different concentrations of Au@TiO_2@DOX NPs at 37 °C for 24 h in a humidified 5% CO_2 atmosphere. For *in vitro* PDT/PTT, HeLa cells were incubated with the different concentration of Au@TiO_2 core-shell NPs at 37 °C for 4 h under the

same conditions and then irradiated by 635 nm laser (2.0 W/cm^2) for 5 min. For *in vitro* chemothearpy/PDT/PTT, HeLa cells were incubated with the different concentration of Au@TiO_2@DOX NPs (W_{DOX}:$W_{Au@TiO2}$=3) at 37 °C for 4 h under the same conditions and then irradiated by 635 nm laser (2.0 W/cm^2) for 5 min. For *in vitro* zwitterionic therapy/chemothearpy/PDT/PTT, HeLa cells were incubated with different concentrations of P(CitAPDMAEMA)@Au@TiO_2@DOX NPs (W_{DOX}:$W_{Au@TiO2}$=3) prepared using PBS (pH 5.0) at 37 °C for 4 h under the same conditions and then irradiated by 635 nm laser (2.0 W/cm^2) for 5 min. After illumination, the cells were incubated for another 24 h. The culture medium was discarded, and 100 μL of dimethylsulfoxide was added. Absorbance was measured at 570 nm.

Mouse tumor model. In our experiments, female BaLb/c mice were purchased from Nanjing KeyGen Biological Technology Co, Ltd. Animal experiments were performed following protocols approved by Nanjing Normal University Laboratory Animal Center. To develop the tumor model, HeLa cells (1×10^6) in 50 μL PBS were subcutaneously injected into the back or oxter of each mouse. After about one week, the average size of tumor was about 60 mm^3.

Fluorescence and MR imaging. P(CitAPDMAEMA)@Au@TiO_2@DOX NPs were intravenously injected into tumor bearing mice. Fluorescent scans were performed at various time points of post-injection (0, 0.5, and 24 h) by a Maestro 2 Multispectral Small-animal Imaging System. The tumor-bearing mice were sacrifi ced by exsanguinations at 24 h post-injection, and the tumor and major organs were harvested.

Mn@P(CitAPDMAEMA)@Au@TiO_2@DOX NPs dissolved in DI water with different concentrations were scanned by a 3.0 T clinical MRI scanner. T_1 weighted animal MR imaging were performed under the same MR scanner with a special coil designed for small-animal imaging.

Blood circulation. 200 μL of P(CitAPDMAEMA)@Au@TiO_2@DOX NPs was intravenously injected into five mice. At each time point, 20 μL of blood was collected from the mouse and then dissolved in 300 μL of lysis buffer (1% SDS, 1% Triton-100, 40 mM Tris acetate, 10 mM EDTA, and 10 mM DTT). To extract DOX from blood, 300 μL of HCl/isopropanol was added. Then, the mixture was incubated in dark overnight. By centrifuging to obtain the DOX in supernatant, the amount of DOX remaining in blood was determined by fluorescence.

***In vivo* combination therapy.** HeLa tumor-bearing mice were divided into five groups, P(CitAPDMAEMA)@Au@TiO_2@DOX NPs (1), PBS (plus Laser) (2), Au@TiO_2@DOX NPs (plus Laser) (3), Au@TiO_2 core-shell NPs (plus Laser) (4), and P(CitAPDMAEMA)@Au@TiO_2@DOX NPs (plus Laser) (5). In each group, 200 μL of materials at the same concentration of DOX (10 mg/kg) were intravenously injected. After 24 h, the tumors were irradiated by the 635 nm laser for 5 min at the power density of 2.0 W/cm^2. During the laser irradiation, the temperature change of tumors was recorded by an Infrared thermal imaging camera. After treatment, the size change of tumor was monitored by digital caliper to record the lengths and widths every two days for two weeks. The tumor volumes were calculated by length× width^2/2. After 14 d treatment, the typical organs (heart, liver, spleen, lung, and kidney) and tumor tissues of the mice in each group were dissected for further histology analysis. The blood sample were separated from the bodies to measure the blood biochemical index.

Supporting Figures

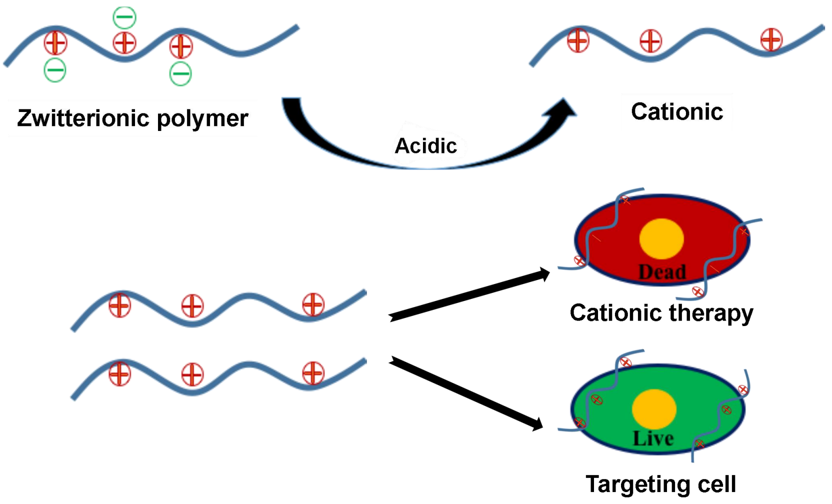

Scheme S1. Schematic illustration of the P(CitAPDMAEMA) for binding to cancer cells and inhibiting cancer cells growth.

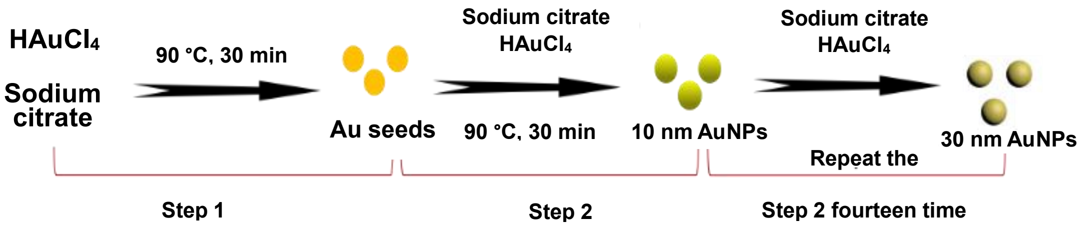

Scheme S2. Scheme illustration of the synthesis process of the AuNPs.

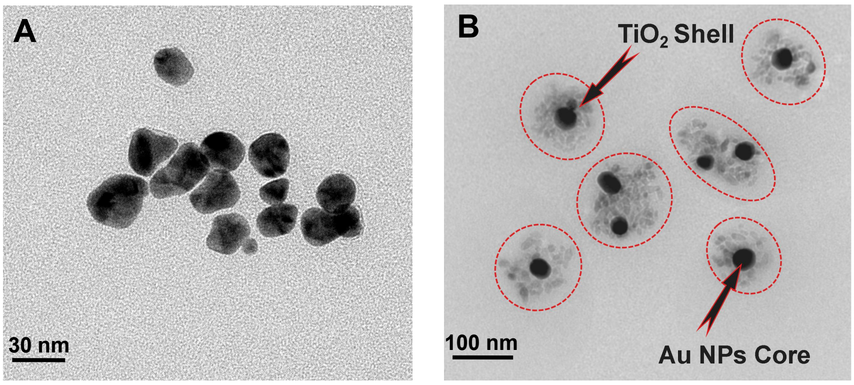

Figure S1. TEM images of (A) AuNPs and (B) Au@TiO_2 core-shell NPs.

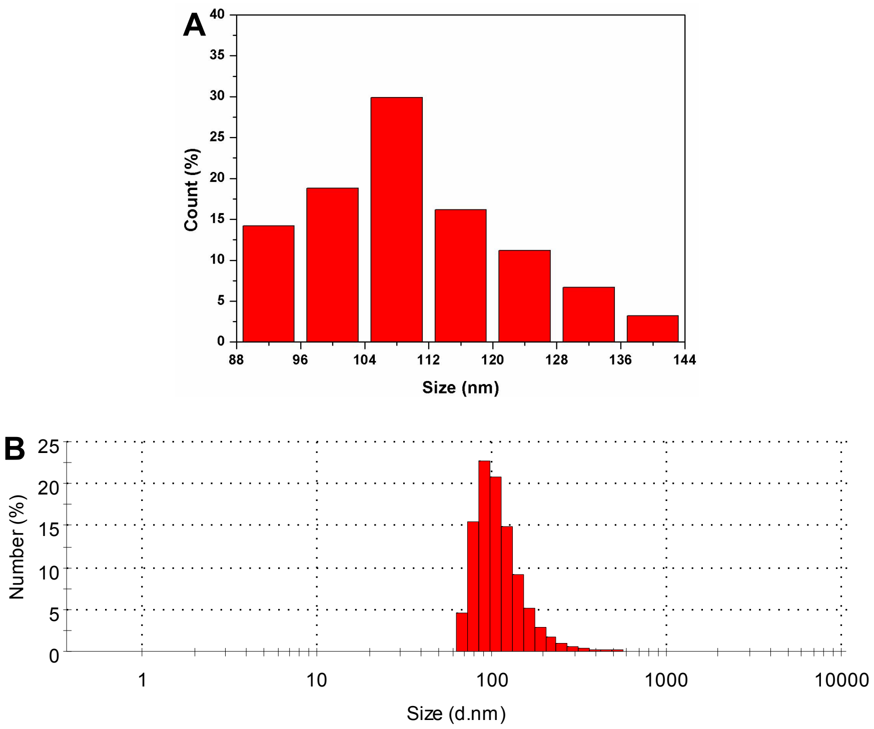

Figure S2. (A) Size distribution and (B) DLS data of Au@TiO_2 core-shell NPs.

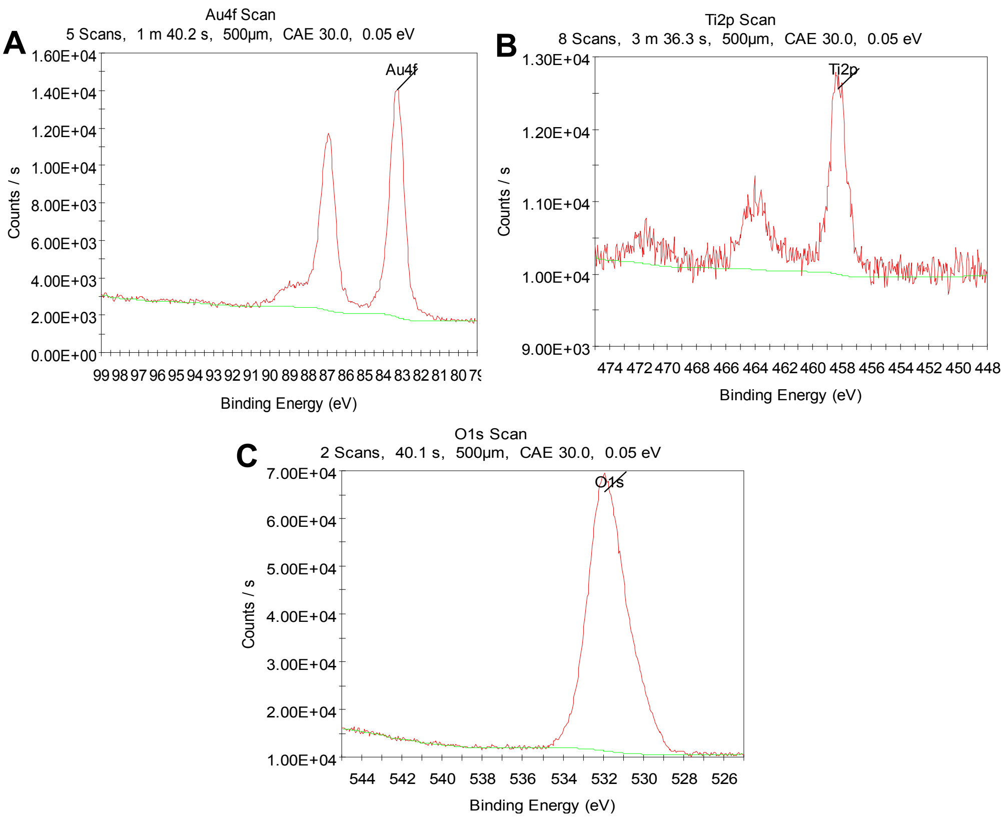

Figure S3. XPS peaks of (A) Au 4f, (B) Ti 2p, and (C) O 1 s of Au@TiO_2 core-shell NPs.

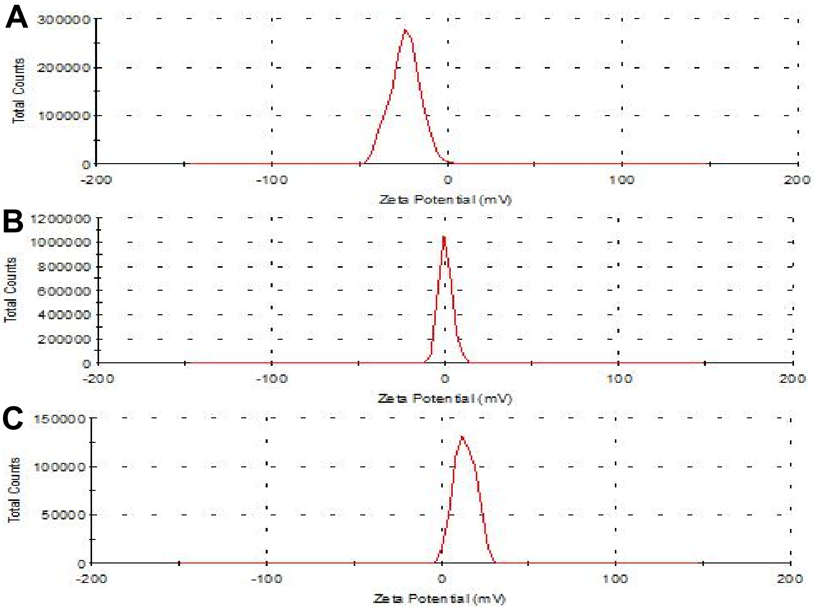

Figure S4. Zeta potential of (A) Au@TiO_2 core-shell NPs, (B) Au@TiO_2@DOX NPs, and DOX (C).

2-(N-Boc-3-aminopropyl-N,N-dimethylammonium)-ethyl methacrylate (BocAPDMAEMA) was obtained by conjugation of 2-(dimethylamino)ethyl methacrylate with N-Boc-3-bromopropylamine. The Boc-protected homopolymer poly(2-(N-Boc-3-aminopropyl-N,N-dimethylammonium)ethyl methacrylate) (P(BocAPDMAEMA)), was synthesized by BocAPDMAEMA using 4-cyanopentanoic acid dithiobenzoate as chain transfer agent [2]. Then, P(BocAPDMAEMA) was confirmed ^{1}H and C^{13} NMR spectroscopy (Figure S5A-B). Finally, P(APDMAEMA) was modified using citraconic anhydride to obtain the poly(N'-citraconyl-2-(3-aminopropyl-N,N-dimethylammonium)ethyl methacrylate) [P(CitAPDMAEMA)], with 100% of the primary amine groups in P(APDMAEMA) converted into amides, which was confirmed by ^{1}H NMR spectroscopy (Figure S5C).

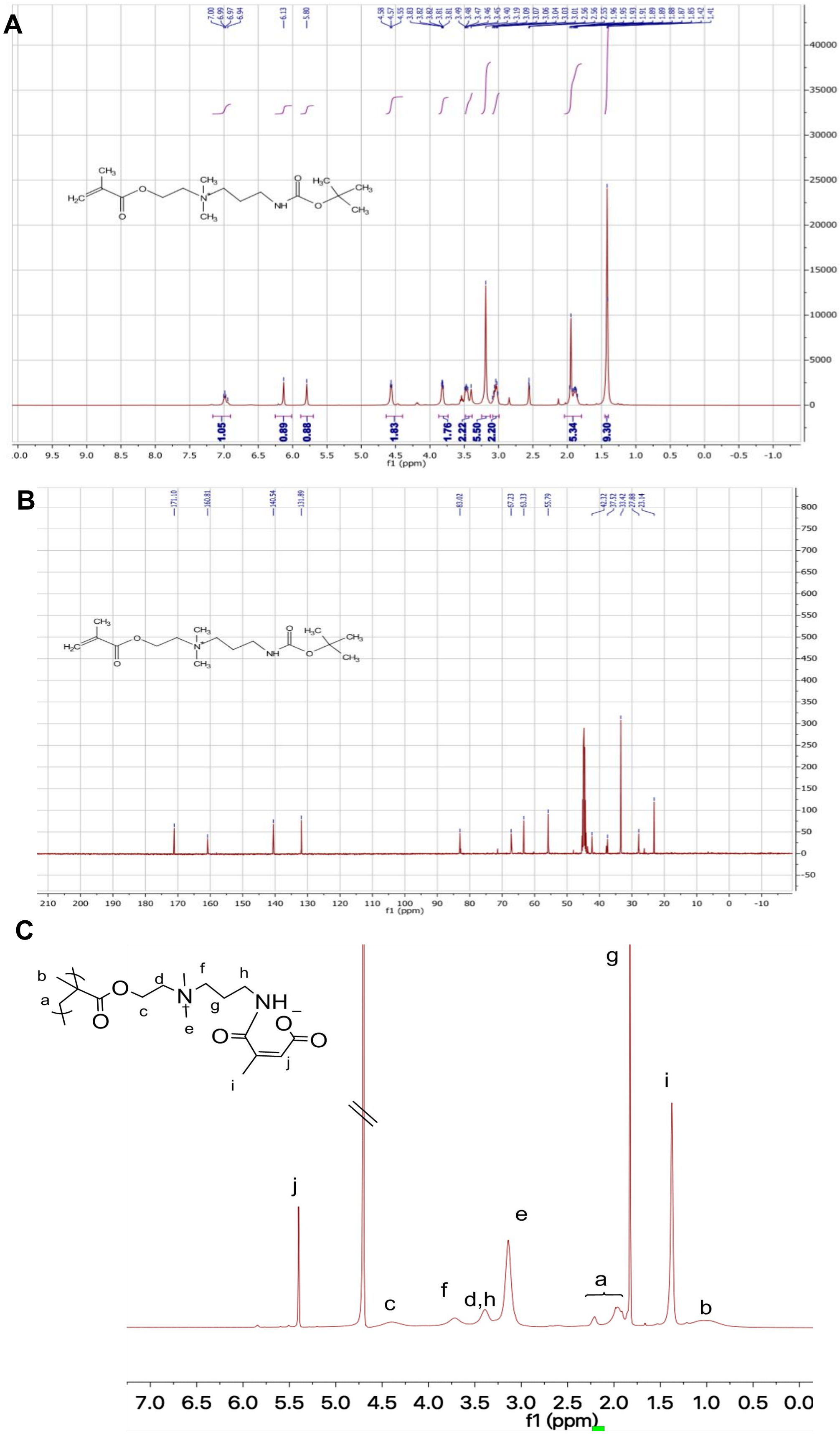

Figure S5. (A) ^{1}H and (B) ^{13}C NMR spectra of P(BocAPDMAEMA). (C) ^{1}H NMR spectra of P(CitAPDMAEMA).

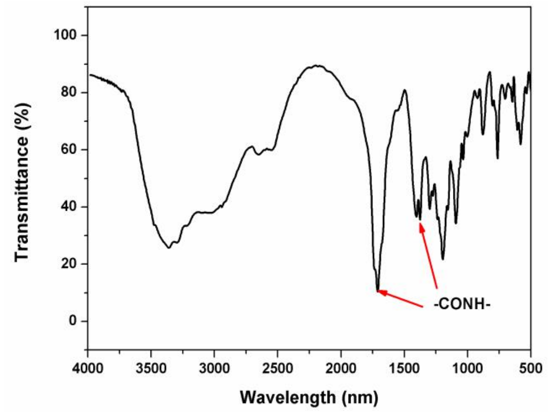

Figure S6. FT-IR spectra of P(CitAPDMAEMA)@Au@TiO_2@DOX NPs.

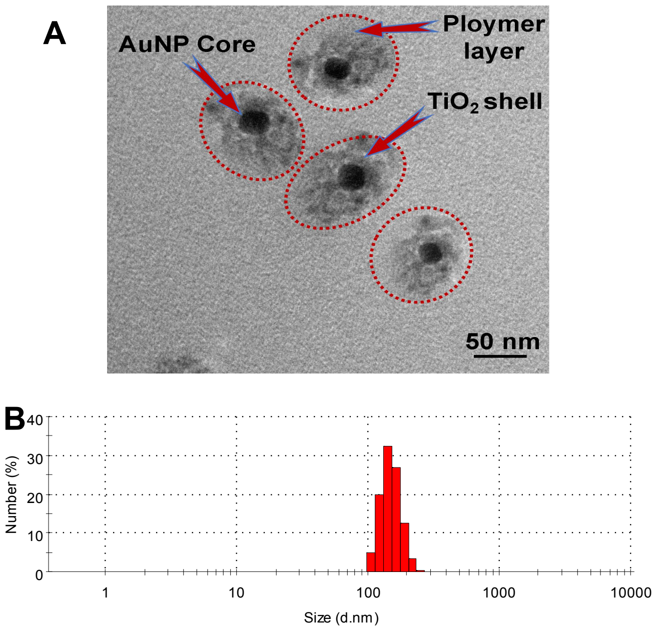

Figure S7. (A) TEM images and DLS of P(CitAPDMAEMA)@Au@TiO_2@DOX NPs.

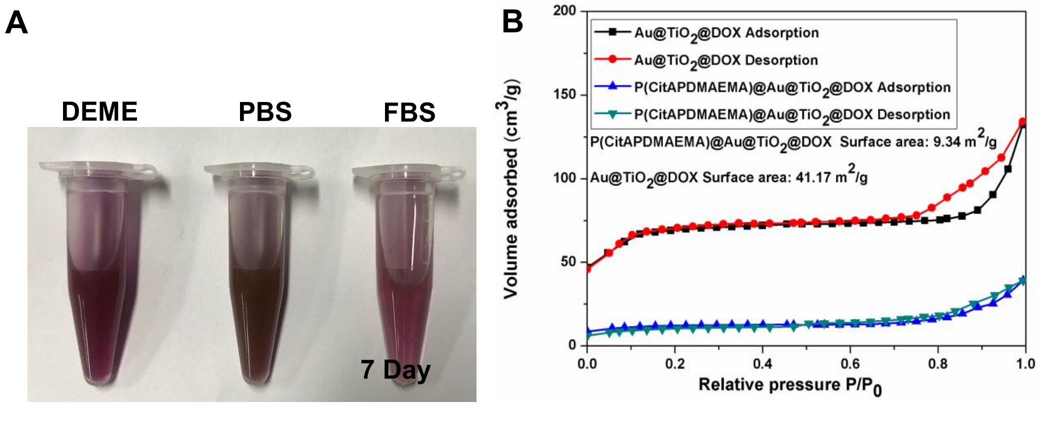

Figure S8. (A) Images of P(CitAPDMAEMA)@Au@TiO_2@DOX NPs in different media. (B) N_2 adsorption-desorption isotherms of P(CitAPDMAEMA)@Au@TiO_2@DOX NPs and Au@TiO_2@DOX NPs.

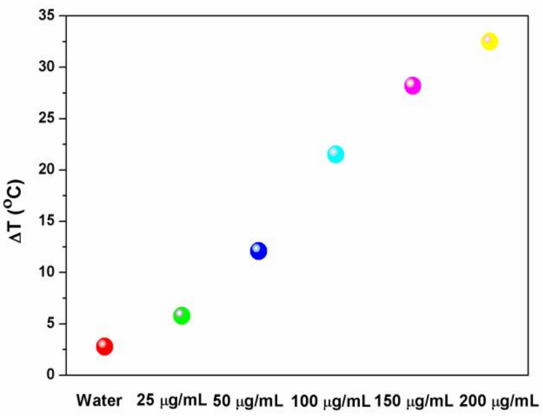

Figure S9. The changes in temperatures at varied concentrations of P(CitAPDMAEMA)@Au@TiO_2@DOX NPs.

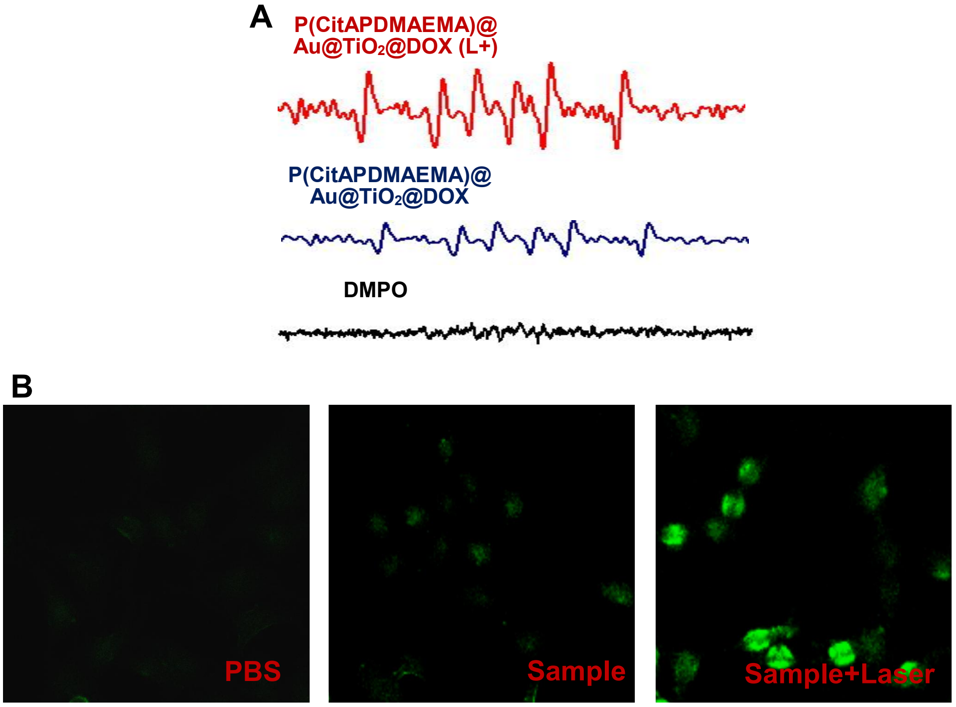

Figure S10. (A) EPR spectra (DMPO/·OOH) of P(CitAPDMAEMA)@Au@TiO_2@DOX NPs with or without laser irradiation. (B) Fluorescent images of cells with different formulation using H2DCFHDA as probe.

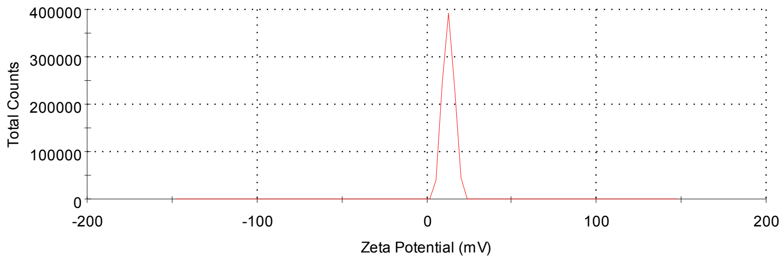

Figure S11. Zeta potential of P(CitAPDMAEMA)@Au@TiO_2@DOX NPs after hydrolysis.

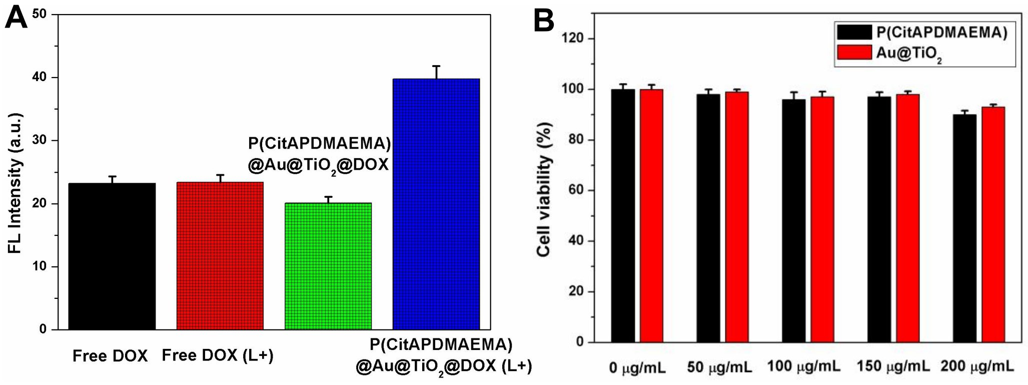

Figure S12. (A) Fluorescence intensity of HeLa cells incubated with free DOX and P(CitAPDMAEMA)@Au@TiO_2@DOX NPs with or without laser irradiation. (B) *In vitro* cell viability of HeLa cells incubated with P(CitAPDMAEMA) and Au@TiO_2 core-shell NPs at different concentrations for 24 h at 37 °C.

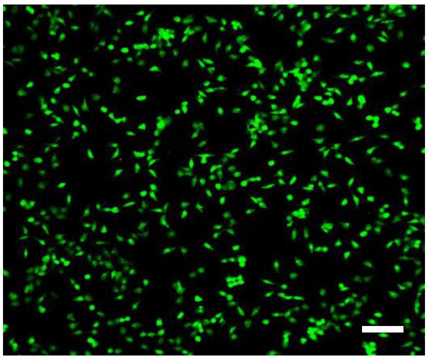

Figure S13. Fluorescence microscopy images of HeLa cells stained with Live/Dead kit after the treatments of P(CitAPDMAEMA) under pH 7.4. Scale bar: 100 µm

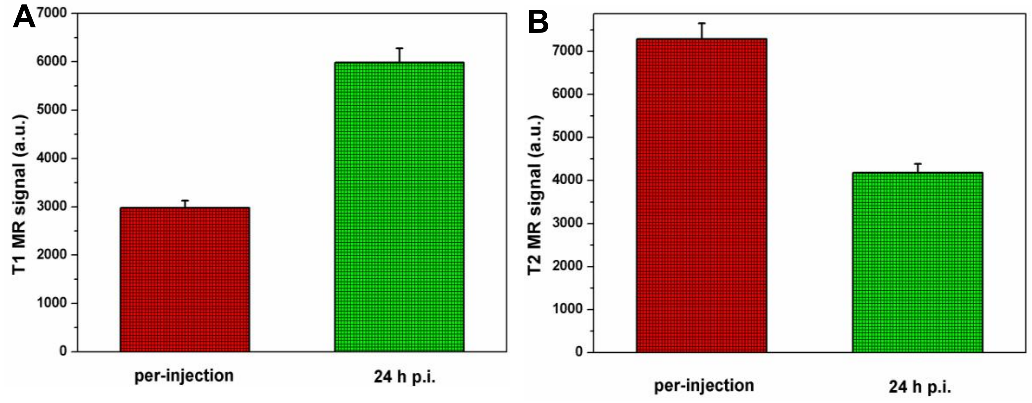

Figure S14. (A) T_1 and (B) T_2 weighted MR signals of untreated and 24 h post injection tumors.

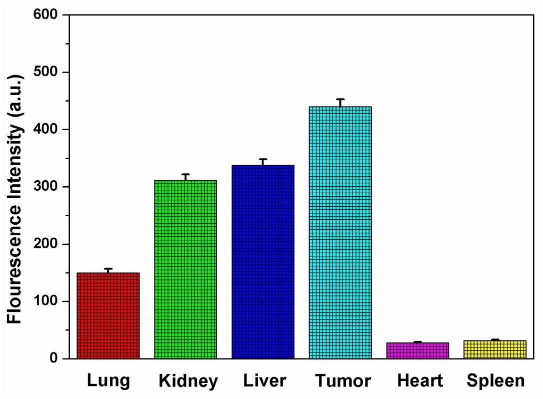

Figure S15. Fluorescence distribution of P(CitAPDMAEMA)@Au@TiO_2@DOX in major organs excised from mice 24 h post injection.

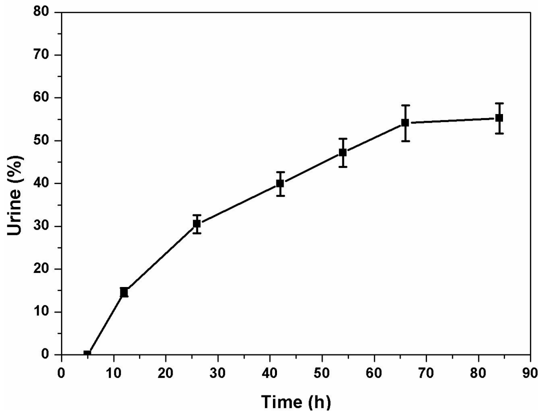

Figure S16. Cumulative excretion of urine at different time points. The Au element content in the urine was measured by an ICP-MS.

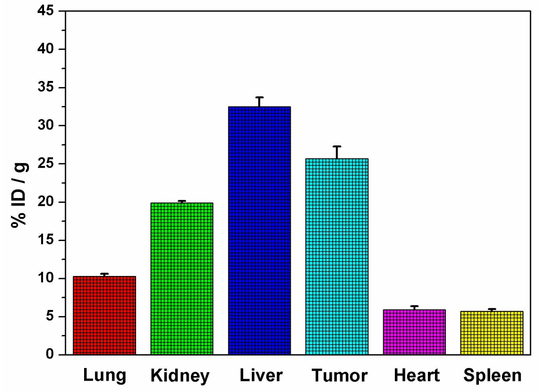

Figure S17. Biodistribution of P(CitAPDMAEMA)@Au@TiO_2@DOX at 24 h after intravenous injection in mice.

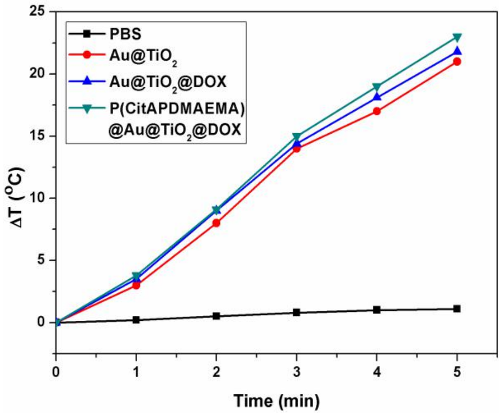

Figure S18. Heating curve of the four laser-irradiated groups.

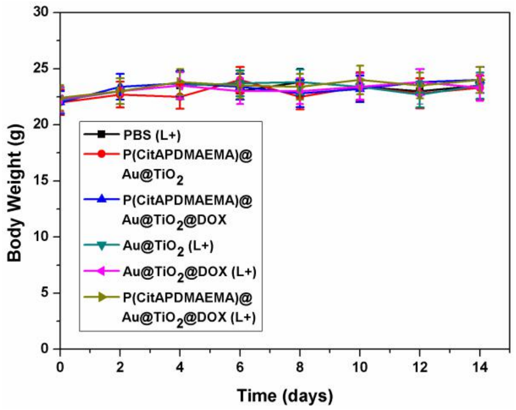

Figure S19. Body weight curves normalized to day 0 for different groups during the experiments.

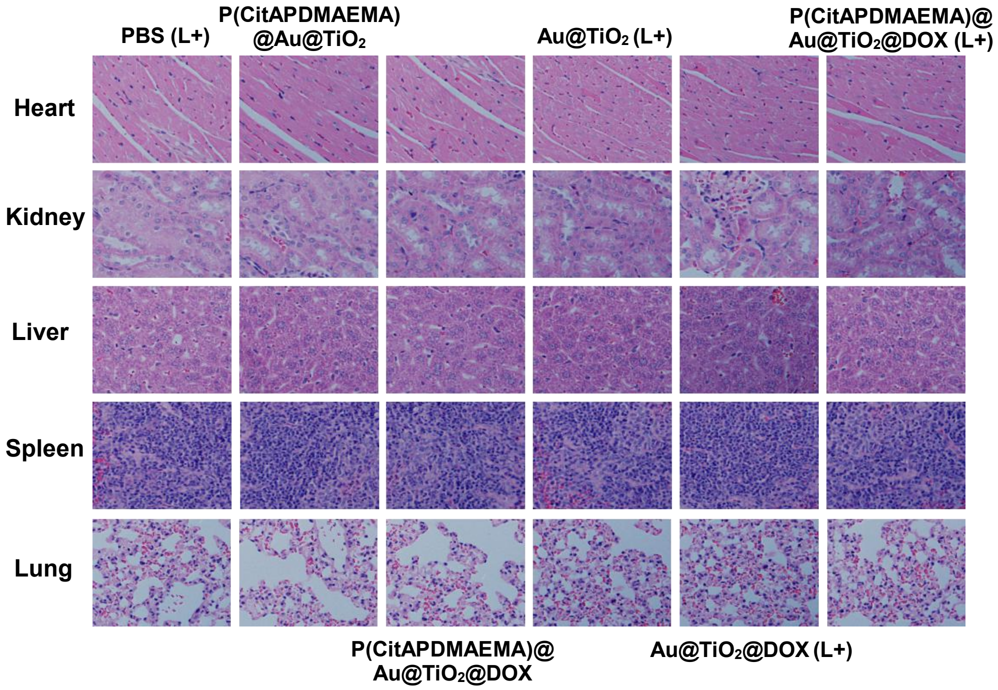

Figure S20. Histological H&E staining of various organs from different groups.

References

1. Sun H, He Q, She P, Zeng S, Xu K, Li J, et al. One-pot synthesis of Au@TiO2 yolk-shell nanoparticles with enhanced photocatalytic activity under visible light. J Colloid Interface Sci. 2017; 505: 884-91.
2. Liu P, Xu G, Pranantyo D, Xu LQ, Neoh K-G, Kang E-T. pH-Sensitive Zwitterionic Polymer as an Antimicrobial Agent with Effective Bacterial Targeting. ACS Biomater Sci Eng. 2017; 4: 40-6.
